# Groundwater releases CO_2_ to diverse global coastal ecosystems

**DOI:** 10.1126/sciadv.adr3240

**Published:** 2025-01-10

**Authors:** Aprajita S. Tomer, Tristan McKenzie, Claudia Majtényi-Hill, Alex Cabral, Yvonne Y. Y. Yau, Mitchell Call, Xiaogang Chen, Rogger E. Correa, Kay Davis, Luke Jeffrey, Mahmood Sadat-Noori, Douglas Tait, Jackie Webb, Damien T. Maher, Linnea Henriksson, Stefano Bonaglia, Shibin Zhao, M. Bayani Cardenas, Isaac R. Santos

**Affiliations:** ^1^ Department of Marine Sciences, University of Gothenburg, Gothenburg, Sweden.; ^2^ National Marine Science Centre, School of Environment, Science and Engineering, Southern Cross University, P.O. Box 4321, Coffs Harbor, NSW 2450, Australia.; ^3^ Faculty of Science and Engineering, Southern Cross University, Lismore, NSW 2480, Australia.; ^4^ Key Laboratory of Coastal Environment and Resources of Zhejiang Province, School of Engineering, Westlake University, Hangzhou 310024, China.; ^5^ Forestry Corporation of NSW, Coffs Harbor, NSW 2450, Australia.; ^6^ Corporacion Merceditas, 050021 Medellin, Antioquia, Colombia.; ^7^ Australian Institute of Marine Science, Indian Ocean Marine Research Centre, Crawley, WA, Australia.; ^8^ College of Science and Engineering, James Cook University, Townsville, QLD 4811, Australia.; ^9^ School of Agriculture and Environmental Science, University of Southern Queensland, Toowoomba, QLD 4350, Australia.; ^10^ Frontiers Science Centre for Deep Ocean Multispheres and Earth System, and Key Laboratory of Marine Chemistry Theory and Technology, Ministry of Education, Ocean University of China, Qingdao 266100, China.; ^11^ Laboratory for Marine Ecology and Environmental Science, Qingdao Marine Science and Technology Center, Qingdao 266100, China.; ^12^ Department of Earth and Planetary Sciences, Jackson School of Geosciences, The University of Texas at Austin, Austin, TX, USA.

## Abstract

Coastal ecosystems play a major role in marine carbon budgets, but substantial uncertainties remain in the sources and fluxes of coastal carbon dioxide (CO_2_). Here, we assess when, where, and how submarine groundwater discharge (SGD) releases CO_2_ to shallow coastal ecosystems. Time-series observations of dissolved CO_2_ and radon (^222^Rn, a natural groundwater tracer) across 40 coastal systems from 14 countries revealed large SGD-derived CO_2_ fluxes. The mean groundwater partial pressure of CO_2_ was 35 times higher than surface seawater. The mean SGD-derived CO_2_ flux was 148 ± 226 millimoles per square meter per day (mmol m^−2^ day^−1^), resulting in a mean water-air CO_2_ flux of 80 ± 133 mmol m^−2^ day^−1^. Tidal rather than diel cycles drove CO_2_ enrichment in most ecosystems. Tidally driven SGD was the primary CO_2_ source in mangroves, salt marshes, tidal flats, estuaries, and canals. Overall, we expand current knowledge of marine carbon cycles by demonstrating SGD as an important source of CO_2_ that requires inclusion in coastal carbon budgets.

## Teaser

Groundwater is a hidden, steady source of CO_2_ to coastal waters.

## INTRODUCTION

The land-ocean interface plays an essential role in modulating Earth’s changing climate and the global carbon budget (*1*). Despite covering a small area globally, this region contributes disproportionately large carbon fluxes. Continental shelves are often atmospheric CO_2_ sinks, whereas rivers and estuaries are usually sources of CO_2_ to the atmosphere (*1*–*3*). Processes such as primary production and respiration, calcification/dissolution, temperature variations, gas exchange, and riverine discharge control CO_2_ dynamics in coastal waters (*4*–*6*). Although these processes have been extensively studied, uncertainties of ~50% persist in key terms of coastal carbon budgets (*1*, *7*). Submarine groundwater discharge (SGD), an important but often overlooked source of nutrients to coastal surface waters, may also be a major source of carbon, further contributing to the large uncertainties in coastal carbon budgets (*8*–*10*).

SGD constitutes all flow of groundwater and porewater from the seabed to the coastal ocean, including terrestrial fresh groundwater inputs and seawater recirculated through sediments (*11*). Fresh groundwater flows are only ~0.6% of the total global freshwater inputs into the ocean but may be important in many estuaries, salt marshes, and coral reefs (*10*). Recirculated seawater is often driven by tides and accounts for >90% of the total SGD in most sites where it has been quantified (*12*–*14*). Total SGD often results in brackish, anoxic water inputs affecting coastal biogeochemistry (*11*, *15*–*17*). For instance, total nitrogen and phosphorus fluxes from SGD exceed river fluxes in ~60% of the sites where both sources have been quantified (*8*). Although the impact of SGD on global nutrient fluxes is now widely recognized (*18*), the contribution from SGD to carbon budgets remains poorly understood. High concentrations of CO_2_ can accumulate in coastal groundwater. This CO_2_ can then be transported to coastal waters via SGD (*19*), eventually be released into the atmosphere (*20*–*22*), modify seawater pH (*23*, *24*), and alter coastal carbon budgets (*25*).

Resolving SGD and coastal carbon budgets is challenging due to spatiotemporal variability and complex driving forces (*9*). Radon (^222^Rn) is an effective SGD tracer because it is enriched in both fresh water and saline water in contact with sediments and is only detectable within a short distance from the source due to its short half-life (3.8 days) and water-air exchange (*24*–*26*). Mass-balance models of ^222^Rn are commonly used to estimate SGD (*26*, *27*). When coupled with CO_2_, continuous, high-resolution measurements of ^222^Rn can be used to explore links between SGD and carbon dynamics in aquatic ecosystems (*28*).

**Fig. 1.**
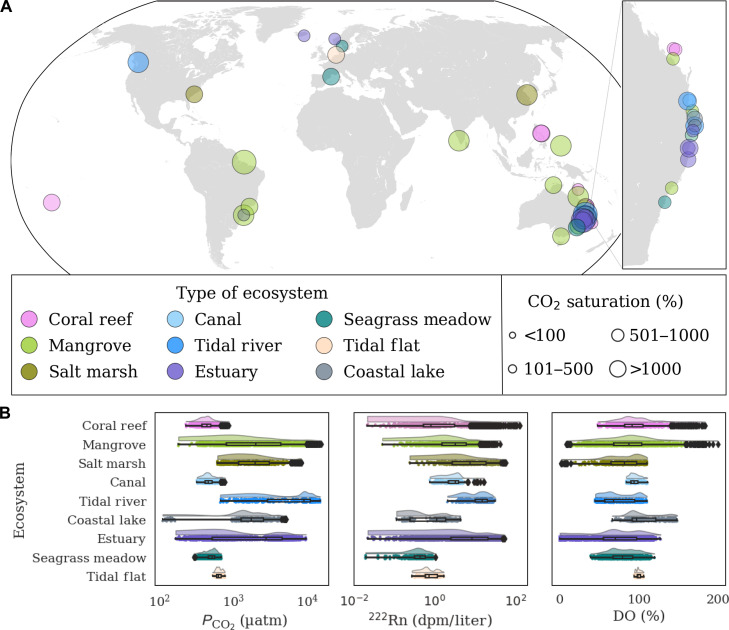
Location of study sites worldwide and general summary of observations. (**A**) The map depicts 40 observation sites across 5 continents and 14 countries. The symbol size represents the mean CO_2_ saturation levels in surface water, and the color scale represents the ecosystem type. The inset highlights locations along the east coast of Australia. Details about specific locations and sources of data are summarized in fig. S1 and table S1. (**B**) The panels show the distribution of *P*co_2_, ^222^Rn, and DO levels in surface water categorized by ecosystem type. Boxplots reveal the median levels of the three parameters along with their interquartile ranges. Jittered dots representing individual data points enable identification of outliers falling outside whiskers. Additional details about surface water observations can be found in table S4.

Here, we address knowledge gaps in coastal carbon budgets by hypothesizing that SGD is an essential source of CO_2_ to coastal waters. We compiled high-resolution dissolved partial pressure of CO_2_ (*P*co_2_), ^222^Rn, and dissolved oxygen (DO; proxy for photosynthesis/respiration) observations from 40 diverse study sites (Fig. 1 and table S1) across nine coastal ecosystems worldwide to assess the relative importance of SGD and biological activity as drivers of *P*co_2_. We also quantify SGD-derived CO_2_ fluxes to coastal waters and examine the contrasting effects of tidal (physical processes) and diel (primary production and respiration) drivers. Although prior regional studies demonstrated that SGD can be a source of CO_2_ to some coastal waters (*29*–*32*), this synthesis shows that SGD is usually a source of CO_2_ to diverse coastal systems worldwide. Overall, our observations challenge the conventional perception that river inputs and respiration are the only major sources of CO_2_ in coastal waters.

## RESULTS AND DISCUSSION

### Widespread oversaturation of CO_2_

Our observations at 40 coastal sites (fig. S1) collected over the span of a decade provide a unique opportunity to investigate coastal CO_2_ sources and dynamics. Coastal surface water *P*co_2_ levels were up to 17 times higher than atmospheric levels, indicating oversaturation of CO_2_ in all ecosystems (Fig. 1). CO_2_ oversaturation was substantial in tidal rivers (~1900%), salt marshes (~580%), and mangroves (~450%), whereas coral reefs and seagrass meadows exhibited relatively lower saturation levels of ~110% (table S2). Oversaturation of CO_2_ can result from the transfer of dissolved organic (*33*) and inorganic carbon (*34*) via SGD to coastal waters, from river inputs, and local respiration. Decomposition of organic matter is enhanced in nutrient-enriched groundwater, adding to the release of CO_2_ from coastal aquifers (*35*, *36*). Relatively lower CO_2_ oversaturation levels occurred in ecosystems with high DO in their water column due to intense primary production (Fig. 1).

Coastal groundwater exhibited, on average, 35 times greater *P*co_2_ than coastal surface water (fig. S2). Highly productive, shallow ecosystems such as canals and estuaries exhibited ~90 times greater *P*co_2_ in groundwater than surface water. Organic-rich blue carbon ecosystems, namely mangroves and salt marshes, along with coral reefs and coastal lakes had moderate enrichment with groundwater *P*co_2_ being ~25 times greater than surface waters. Groundwater CO_2_ enrichment is attributed mostly to the accumulation of aerobic and anaerobic respiration products following microbial breakdown of organic matter (*31*) as well as silicate weathering and carbonate precipitation in groundwater (*37*). In systems such as canals, estuaries, and coral reefs, surface water CO_2_ removal by photosynthesis also enhances the perceived enrichment of groundwater due to an increase in groundwater–to–surface water ratio of *P*co_2_.

All coastal ecosystems were either near-equilibrium or undersaturated in DO (table S2). Coastal lakes, canals, and coral reefs exhibited near-equilibrium oxygen saturation. However, tidal ecosystems, including mangroves, estuaries, salt marshes, and tidal rivers, were undersaturated at ~75%. The oxygen undersaturation is consistent with aerobic respiration in both ground and surface waters using oxygen and producing CO_2_. Thus, SGD enriches CO_2_ in surface waters, bringing these ecosystems toward hypercapnia (abnormally high levels of *P*co_2_) (*38*). In coastal lakes, algal blooms and eutrophication occasionally attenuate hypercapnia by releasing oxygen and consuming CO_2_ during the day, potentially masking SGD-derived CO_2_ inputs (*39*). As expected, groundwaters were undersaturated in DO and approached zero in many cases (table S3). The overall negative correlations between surface water radon and DO (fig. S3) support recent suggestions that coastal seawater deoxygenation may be partially explained by SGD (*40*).

### Tidally driven SGD as a source of CO_2_

**Fig. 2.**
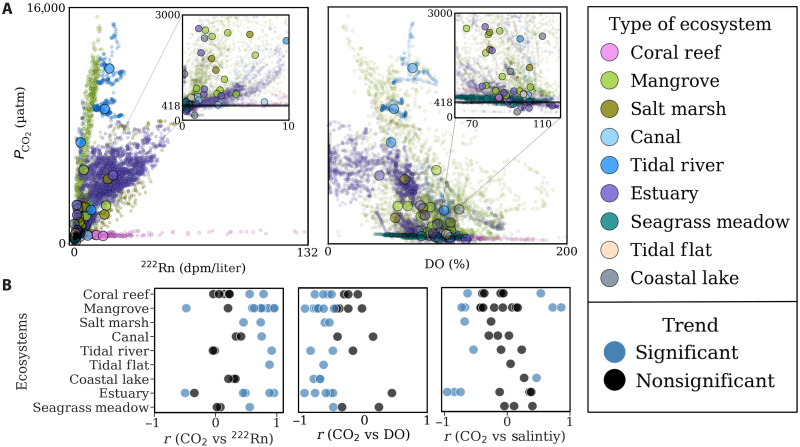
Correlations between *P*co_2_ and the proxies for groundwater (^222^Rn) and productivity (DO). (**A**) Significant positive correlations were found between *P*co_2_ and ^222^Rn in 25 of 40 sites (see also figs. S4 and S5). The points above the black line in the insets depict surface water *P*co_2_ levels exceeding atmospheric *P*co_2_. The small points represent individual time-series observations, whereas the larger points represent site-specific means. (**B**) CO_2_ versus ^222^Rn, DO, and salinity *r* values for each site (refer to figs. S4 and S5 and table S4).

SGD traced by ^222^Rn was a major source of CO_2_ in mangroves, salt marshes, tidal flats, estuaries, and canals (Fig. 2 and fig. S4). Contrastingly, biological activity (traced by oxygen) had a greater influence on CO_2_ in shallow coastal systems such as coral reefs and coastal lakes (fig. S5). These results were further supported by a driver-response relationship using a cumulative sum (CUSUM) analysis (fig. S6) as well as a significant (*P* < 0.05) positive correlation between CO_2_ and ^222^Rn and a negative correlation of CO_2_ with DO (Fig. 2). The relationship between *P*co_2_ and salinity was either absent or weak (table S4). Freshwater sources include direct rainfall, rivers, and fresh groundwater. Rainfall cannot be a CO_2_ source because it has *P*co_2_ values in equilibrium with the atmosphere, much lower than our observations in nearshore seawater. The low or negligible correlations between the groundwater tracer ^222^Rn and salinity suggest that recirculated seawater, not fresh groundwater, was the major source of ^222^Rn. All sites were located nearshore by potential groundwater sources (fig. S1). The often-insignificant salinity versus *P*co_2_ correlations (Fig. 1) imply that seawater recirculation in sediments rather than freshwater inputs drove CO_2_ supersaturation during the observation periods.

The driver-response (CUSUM) analysis (fig. S6) supports the interpretation from correlations (Fig. 2) to suggest that SGD drives CO_2_ dynamics in tidally dominated ecosystems. Radon activities of >5 dpm/liter in these ecosystems explained increasing *P*co_2_ (fig. S6). Aerobic respiration traced by oxygen was the dominant process driving CO_2_ inputs in shallow, highly productive ecosystems with low ^222^Rn activities of <2 dpm/liter and high oxygen. Similarly, undersaturated DO values <75% are coupled to enhanced CO_2_. Lower DO values are expected near groundwater sources (*40*, *41*). Temperature plays a major role in open-ocean *P*co_2_ distribution with supersaturated warm tropical waters acting as sources to the atmosphere and undersaturated cold polar waters acting as a sink (*42*). As expected, we observed higher *P*co_2_ values in warm mangrove waters and low values in cold waters in Sweden and Iceland (fig. S7), but the trends were highly scattered compared to other *P*co_2_ controls.

**Fig. 3.**
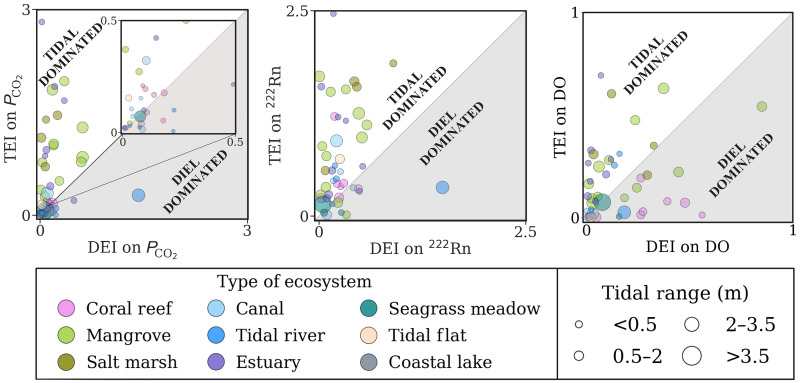
Comparative analysis of tidal and diel effects. The effects on *P*co_2_, ^222^Rn, and DO in surface water, captured during peak hours (fig. S10) of diel and tidal cycles across 40 study sites. The tidal and diel effect indices (TEI and DEI, respectively) describe the relative effect of tidal and diel processes. The white area depicts the region primarily influenced by tides, whereas the gray area represents the region primarily influenced by diurnal cycles. Each point represents an individual study location. The size of the points varies with the tidal range (m) for each site. The color scheme represents the ecosystem type. Two outliers were excluded.

Tides can modulate SGD and CO_2_ in coastal ecosystems on timescales of hours (low-high and ebb-flood tidal cycles; Fig. 3 and figs. S8 to S10) to weeks (spring-neap cycles; fig. S11) (*13*, *43*). Tidal pumping refers to the infiltration of seawater into coastal aquifers at high tide and subsequent discharge at low tide (*44*, *45*), releasing CO_2_-enriched groundwater to the coastal ocean. The maximal hydraulic gradient between coastal aquifers and the ocean typically occurs at low tide (*46*, *47*) during spring tides (*48*, *49*), increasing SGD (*47*). Delayed groundwater discharge (*50*) may generate greater radon and CO_2_ responses in surface water during the early flood tide than during the ebb tide at a similar water level (fig. S10). Tides also control the duration and frequency of intertidal zone inundation in coastal wetlands, enhancing the area available for sediment-water exchange (*43*) and potentially enhancing both radon and CO_2_ in receiving surface waters following tidal flooding of large intertidal areas during spring tides (*21*, *51*, *52*). Overall, tidal pumping occurs on multiple timescales and delivers a radon signal from sediments and a carbon dioxide signal from organic matter respiration, enhancing CO_2_ in tidal environments.

On hourly timescales, the tidal influence on CO_2_ was five times greater than that of diurnal day-night cycles in canals, mangroves, estuaries, salt marshes, tidal rivers, and tidal flats (Fig. 3). Tidally driven SGD and CO_2_ enhancement were also apparent from the inverse correlation between CO_2_ and water depth in ~50% of the coastal systems (table S4). Diurnal rhythms governed CO_2_ dynamics in nontidal coastal lakes and some coral reefs (fig. S8). Light and dark periods regulate biological activity (e.g., photosynthesis and respiration) (*53*). Prior time-series observations in nearshore waters have primarily focused on diurnal patterns concluding that CO_2_ is strongly governed by biological activity (*54*, *55*). Our analysis covering multiple tidal timescales (figs. S8 to S11) reveals that tidally driven SGD was also a key driver of CO_2_ dynamics in nearshore waters.

We compare surface water time-series observations to groundwater end-member concentrations to further assess whether SGD was a major source of CO_2_ to surface waters. Projecting the regression of *P*co_2_ versus ^222^Rn in surface waters to the groundwater end-member (fig. S12) revealed that the SGD proxy ^222^Rn best explains CO_2_ enrichment in eight of nine ecosystem types. If we assume that groundwater is the only source of CO_2_ to surface waters, then mixing CO_2_-enriched groundwater with depleted surface seawater should result in a projection toward the groundwater end-member. A nonlinear relationship implies that other drivers influence CO_2_ dynamics. This was the case for coastal lakes, where the projected CO_2_ deviated by ~370% from the predicted groundwater end-member (table S2), implying major CO_2_ sources beyond groundwater.

### SGD versus water-air CO_2_ fluxes

This study compiled the largest available dataset on SGD-derived CO_2_ in coastal ocean, addressing a major gap in coastal carbon assessments (*3*). Radon mass balances revealed that mean groundwater discharge rates were the lowest in the two nontidal coastal lakes and highest in a coral reef receiving direct hydrothermal inputs (table S5). Uncertainties in radon mass-balance models are often high due to natural variability in groundwater discharge rates and the multiple terms of radon mass-balance models (*27*, *56*, *57*). The largest uncertainties exceeding 100% occurred when groundwater discharge rates approached zero in a coastal lake, and relatively lower uncertainties occur when SGD rates are larger than 20 cm/day.

**Fig. 4.**
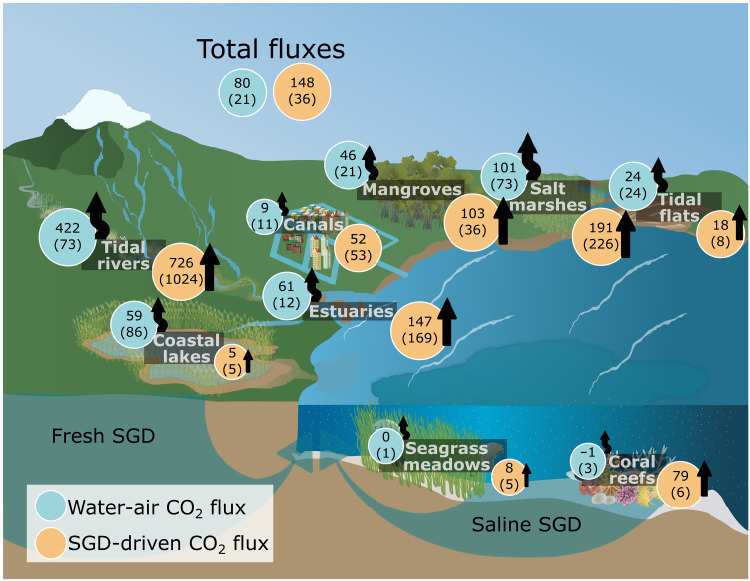
Summary of SGD-derived and water-air CO_2_ fluxes (mmol m^−2^ day^−1^). Mean (median) values depicted for SGD-derived and water-air CO_2_ fluxes for different ecosystem types. SGD-derived CO_2_ fluxes received by the surface waters are in brown circles. Water-air CO_2_ fluxes are in blue circles. Arrows represent SGD-derived CO_2_ fluxes (bottom) and water-air CO_2_ fluxes (top). The circle and arrow sizes qualitatively represent the value of the flux (refer to tables S2 and S5). Icons from T. Saxby retrieved from https://ian.umces.edu/media-library/.

Multiplying these site-specific radon-derived SGD rates (table S5) by the CO_2_ groundwater end-member (table S3) led to a mean SGD-derived CO_2_ flux of 148 ± 226 mmol m^−2^ day^−1^ across all ecosystems. Uncertainties propagations range from <10% in three coral reefs to >100% in three estuaries due to large natural variability in groundwater CO_2_ concentrations. SGD-derived CO_2_ fluxes were highest in ecosystems such as tidal rivers (Fig. 4) due to high CO_2_ enrichment in groundwater (*58*). In contrast, coastal lakes and seagrass meadows had the lowest SGD-derived CO_2_ fluxes. Tidal pumping enhances SGD in nearshore ecosystems with nearby tidal flats and beaches (*21*, *46*). Seagrass meadows efficiently consume seawater CO_2_ through photosynthesis (*59*), hindering CO_2_ accumulation into the water column from sources such as SGD. Moreover, subtidal seagrass meadows have reduced influence of tidal pumping, lowering SGD fluxes.

The mean CO_2_ water-air flux in all ecosystems was 80 ± 133 mmol m^−2^ day^−1^ with 85% of the sites releasing CO_2_ to the atmosphere (table S2). Water-air CO_2_ fluxes for coral reefs and seagrass meadows were near equilibrium when averaged over complete diel and tidal cycles. Coastal lakes were the only ecosystem type where the CO_2_ water-air flux substantially exceeded groundwater-derived fluxes likely due to low groundwater inputs and other CO_2_ sources (Fig. 4). Tidal rivers and salt marshes exhibited the highest CO_2_ water-air fluxes that exceed previously established global averages (*5*, *30*). Notably, the mean water-air CO_2_ flux in salt marshes was ~7 times greater than the global average reported in the literature (*60*). Other ecosystems had mean water-air CO_2_ flux rates comparable to those reported in the literature (*61*–*63*). For instance, a mean water-air CO_2_ flux of 46 ± 54 mmol m^−2^ day^−1^ was observed in our mangrove sites, which is comparable to global mean fluxes ranging from 43 to 72 mmol m^−2^ day^−1^ (*64*–*67*). Observed CO_2_ water-air fluxes in this study are partly attributed to SGD given the proximity of surface water measurements to SGD sources (fig. S13) (*1*, *68*).

The median zero-radon intercept from the ^222^Rn versus CO_2_ scatterplots (fig. S4) was ~411 μatm (mean = 1200 ± 2290 including freshwater outliers), a value remarkably close to atmospheric equilibrium (~400 to 418 μatm depending on the year of observations) when no CO_2_ sources are present in surface waters. The widespread surface water CO_2_ enrichment at high ^222^Rn concentrations further demonstrates that SGD saturates water column CO_2_ and prevents further absorption of atmospheric CO_2_ in highly productive coastal waters. The delivery of SGD into high alkalinity seawater will lead to buffering of a fraction of SGD-derived CO_2_ input to form bicarbonate ions (*69*). Ecosystems with high respiration rates and lower buffering capacity such as tidal rivers and coastal lakes deviate from this pattern (*70*) and are clear outliers.

### Summary and implications

Our multiple lines of evidence including a driver-response analysis (fig. S6), correlation analysis (Fig. 2), projections of surface water observations to groundwater end-members (fig. S12), groundwater observations (fig. S2), and local-scale flux estimates (Fig. 4) converge to the conclusion that groundwater enriched surface water CO_2_ in all tidal, nearshore shallow ecosystems. Across all globally distributed ecosystems, mean SGD-derived CO_2_ and water-air CO_2_ fluxes were 148 ± 226 and 80 ± 133 mmol m^−2^ day^−1^, respectively. Groundwater-derived CO_2_ may drive local coastal acidification due to increased CO_2_ inputs, which is especially important in sensitive ecosystems like coral reefs (*71*). Tides modulated nearshore seawater CO_2_ enrichment via tidal pumping enhancing groundwater exchange across the land-ocean continuum.

Our observations add conceptual insight into coastal carbon cycle investigations. Existing coastal carbon budgets emphasize river inputs, transformations within estuaries, and exchange with intertidal wetlands (*72*). Intertidal wetlands are SGD and CO_2_ exchange hotspots. Tidally driven groundwater exchange in intertidal mangrove and salt marsh sediments accelerates organic matter respiration (*43*), explaining the particularly high fluxes observed in those ecosystems. Intertidal wetlands often cover small areas of river catchments but may account for over 50% of the total terrestrial carbon fluxes reaching the ocean as observed in Australia (*73*). Groundwater-derived CO_2_ fluxes in mangroves and salt marshes return 12 to 16% of the total primary productivity to the atmosphere (*7*), somewhat minimizing the potential for carbon sequestration in sediments. Our estimated groundwater-derived CO_2_ fluxes are site specific and can contribute toward reducing uncertainties in local carbon budgets. Although our observations imply widespread impacts of SGD on coastal seawater CO_2_ enrichments, the available datasets are not enough to allow for reliable global extrapolations. The best-sampled ecosystems are mangroves (*n* = 12 sites) and coral reefs (*n* = 7). The mean SGD-derived CO_2_ fluxes in those ecosystems range from 1 to 540 mmol m^−2^ day^−1^ and have large natural variabilities that create major challenges for reasonably upscaling and extrapolating the data.

This study emphasizes the need for process-based investigations of groundwater-mediated coastal carbon dynamics and highlights the paucity of data in African, Middle Eastern, and European regions. Additional observations are particularly important in data poor ecosystems such as coastal lakes (*n* = 2 sites), salt marshes (*n* = 2), and seagrass meadows (n = 3). Filling these data gaps is crucial to further build confidence in interpretations, enable global-scale extrapolations, and fully quantify the contribution from SGD to global coastal carbon budgets. Overall, our results highlight that coastal carbon budgets and management approaches to maximize coastal carbon sequestration should consider SGD (*74*) alongside better understood carbon sources such as rivers, pelagic respiration, and anthropogenic inputs.

## MATERIALS AND METHODS

### Data acquisition

We compiled high-resolution CO_2_ and ^222^Rn time-series observations from 40 different locations worldwide (Fig. 1, fig. S1, and table S1). The sites represent nine major coastal ecosystem types (coral reef, mangrove, salt marsh, canal, freshwater tidal river, estuary, tidal flat, coastal lake, and seagrass meadow) (fig. S1). The criteria for inclusion of a dataset were availability of observations for the groundwater tracer ^222^Rn (temporal resolution of ~30 min; dpm liter^−1^), *P*co_2_ (temporal resolution of ~10 min; μatm), temperature (°C), and salinity for at least two tidal cycles. Additional parameters include DO (%), wind speed (m/s), and water depth (m). Of 40 locations, 32 datasets were previously published, and 8 datasets are original (table S1). All measurements were aligned so that the time stamps are the same for all variables. After alignment, the data were processed in Python (version 3.10.11). The conceptual model was created with Inkscape (version 0.93).

### Time-series observations

The same sampling methods were used to obtain continuous surface water time-series observations from all 40 sites. ^222^Rn was measured to quantify SGD fluxes. In these studies, CO_2_ and ^222^Rn observations were obtained using a (LI-COR)-RAD7 coupled system (*28*). The LI-COR measures the *P*co_2_, and the RAD7 (Durridge Inc.) measures the ^222^Rn concentration based on its alpha-emitting daughter ^218^Po with precisions of 2 μatm and 10%, respectively (*28*). To obtain high-resolution observations, we pumped surface water into a showerhead air-water equilibrator (RAD AQUA). The air in the headspace of this equilibrator is then circulated through a Drierite desiccant into the calibrated LI-COR and RAD7 setup. The air-water equilibrator and gas detectors were connected in a closed air loop with the gas detectors connected in series.

The LI-COR measures the *P*co_2_ every second. The ^222^Rn concentrations were integrated over 10- to 60-min intervals depending on the concentrations encountered. Time lags of about 10 min are expected for CO_2_ and 30 min for ^222^Rn due to air-water equilibration time and radioactive decay within the air loop (*28*). Corrections were made to adjust for these time lags. The dry molar concentration of CO_2_ was converted to *P*co_2_ using temperature and salinity (*75*). The concentrations of ^222^Rn were calculated from solubility coefficients (*76*).

### Ancillary parameters

Calibrated Hydrolab MS5 optical sensors, miniDOT DO loggers, Hach LDO with luminescent sensors, and Van Essen and Hobo conductivity-temperature-depth sondes were used to measure the DO, depth, temperature, and salinity in surface water. The temporal resolution of observations ranged between 1 and 30 min and was aligned to ^222^Rn time steps. Wind speeds were obtained from weather stations located within 10 km of the observation sites. For sites where wind speeds were not available, we used reanalysis data from ERA5 (*77*). The raw reanalysis data were then processed in MATLAB to obtain hourly wind speeds. Atmospheric CO_2_ levels at each sampling site and date were obtained from historical trends documented by the National Oceanic and Atmospheric Administration (NOAA) (noaa.gov).

### Groundwater end-member sampling

Discrete groundwater samples were collected near the surface water time-series sampling locations as summarized in table S3. The groundwater observations were compared to surface water values and used to estimate local-scale SGD-derived CO_2_ fluxes that can be compared to local water-air CO_2_ fluxes. Groundwater end-members were obtained from a push-point piezometer system or monitoring bores up to ~1 m deep. A peristaltic pump was used to sample the groundwater from bores or push-point piezometer systems (*78*). The tubing and bores were flushed several times prior to sampling. A handheld multiprobe was used to determine the ancillary water quality parameters such as the temperature, DO, and salinity. For analysis of ^222^Rn, groundwater was sampled in a 6-liter high-density polyethylene bottle that was then connected to the RAD7 (*79*). The groundwater CO_2_ values were determined from the head-space equilibration technique and a Picarro G2201-i (*80*) or from the analysis of dissolved inorganic carbon and alkalinity using the CO2Sys program (*81*) with carbonate system constants from Mehrbach *et al.* (*82*) refit by Dickson and Millero (*83*). Groundwater end-members are not available for seven sites, preventing calculations of SGD-derived CO_2_ fluxes at all sites. The groundwater sample size then ranged from 3 to 48 with 20 of 40 sites having >10 samples (table S3).

### CO_2_ flux calculations

Water-air fluxes for CO_2_ (Eq. 1) were calculated using the method described in ref. (*84*)

$$F={\mathrm{kK}}_{0}\Delta P{\text{co}}_{2}$$ (1)

where *F* is the water-air CO_2_ flux, *k* is the gas transfer velocity for CO_2_ (m day^−1^), Δ*P*co_2_ is the water-air gradient of *P*co_2_ (μatm), and *K*_0_ is the solubility coefficient of CO_2_ (mol kg·atm^−1^) determined as a function of temperature and salinity (*85*). The gas velocity was determined using Eq. 2

$$k=0.31{\left(u\right)}^{2}{\left(\mathrm{Sc}/660\right)}^{-0.5}$$ (2)

where *k* is the gas transfer velocity for CO_2_ (m day^−1^), *Sc* is the Schmidt number, and *u* is the wind speed (m/s). This approach assumes that wind is the main driver of turbulence and CO_2_ exchange at the air-water interface. In some cases, currents and tidal variations may enhance turbulence (*86*). Because current observations are not available for most sites, we report conservative, wind-driven CO_2_ fluxes that can be directly compared across multiple ecosystems.

### Radon mass-balance model

Groundwater discharge rates were calculated using ^222^Rn mass-balance models as explained in detail in the local-scale studies listed in table S1 and a recent review paper (*27*). In short, the ^222^Rn mass balance accounts for all or most of the sources and sinks of ^222^Rn in surface waters (Eq. 3). The sources include groundwater inputs, ^226^Ra decay, sediment diffusion, and upstream inputs. The potential sinks include evasion, downstream exports, and ^222^Rn decay (*31*, *87*)

FgwRngw+FupRnup+DdifA+Raλ222 226V=FdownRndown+Rnλ222 222V+Jatm (3)

where *F*_gw_ stands for the groundwater discharge (m^3^/day), *Rn*_gw_ is the mean groundwater end-member concentration (dpm m^−3^), *F*_up_*Rn*_up_ is for the ^222^Rn flux upstream or mixing onshore (dpm day^−1^), *D*_dif_ is the diffusive ^222^Rn flux (dpm m^−2^ day^−1^), *A* is the wetted surface area of the section (m^2^), *V* is the water volume (m^3^), and ^226^*Ra*λ_222_*V* is for the ^222^Rn inputs from ^226^Ra decay (dpm day^−1^). *F*_down_*Rn*_down_ is for the downstream or offshore ^222^Rn flux (dpm day^−1^), ^222^*Rn*λ_222_*V* is the ^222^Rn decay (dpm day^−1^), and *J*_atm_ represents the atmospheric ^222^Rn evasion rate (dpm day^−1^). Minor changes to this ^222^Rn mass-balance approach are required depending on the local geomorphology. For example, tidal creeks in mangroves and salt marshes require the integration of all fluxes over complete tidal cycles (*50*). Straight shorelines result in estimated fluxes over 1-hour time steps using a nonsteady approach (*87*). *J*_atm_ was determined using wind speed (Eq. 4) (*88*)

$$J_{\text{atm}}=k\left(C_{w}-\alpha C_{\text{air}}\right)$$ (4)

where *k* is the gas transfer velocity (m day^−1^) at the boundary of air-water that has been corrected for the Schmidt number for ^222^Rn at in situ temperature and salinity, *C*_w_ is the concentration of ^222^Rn in water, *C*_air_ is the concentration of ^222^Rn in the atmosphere, and α is the Ostwald solubility.

### Data analysis and interpretation

Pearson correlation coefficients were calculated between *P*co_2_ versus ^222^Rn, DO, and salinity. A relationship was considered at least moderately correlated if −0.5 ≥ *r* ≥ 0.5. Confirmation of driver-response relationships between *P*co_2_, ^222^Rn, and DO was conducted using a CUSUM driver-response analysis (*89*). In this analysis, potential drivers (^222^Rn and DO) are paired with a response variable (*P*co_2_). The drivers are then sorted into ascending order. The CUSUM for the response variable (*s_i_*) is calculated using Eq. 5, where *z_i_* represents the *i*th value in the standardized dataset

$$s_{i}=z_{i}+s_{i-1}$$ (5)

The slope of the CUSUM trend relative to the driving variable can indicate increasing, decreasing, or no effect relationships between driver and response variables.

We also assessed whether *P*co_2_ was driven by diel or tidal (low tide versus high tide) cycles (fig. S11). A strong diel cycle (day versus night) signature implies that photosynthesis and respiration are the primary controls on *P*co_2_ (*39*). In comparison, tidal cycle predominance (low tide versus high tide) implies that hydraulic head gradients between groundwater and the ocean (steeper at low tide) are the main driver of *P*co_2_ dynamics (*50*). We calculated diel and tidal indices for each successive low-high tide and day-night cycle (Eq. 6 and fig. S9). Data from every location were selected within the range of ±1 hour from the peak hour of each low tide, high tide, day (12:00), and night (00:00) (fig. S10) with respect to the local time zone. We then calculated the mean values of DO saturation (%), ^222^Rn (dpm/liter), salinity, and *P*co_2_ (μatm) for each data bracket. Once the data were selected, two new coefficients were computed to quantify the impact of tidal and diel cycles on the parameter (η)

$$\begin{array}{c} \text{Tidal effect index}\;\left(\text{TEI}\right)=\frac{\sqrt{{\left(\eta_{H}-\eta_{L}\right)}^{2}}}{\bar{\eta }};\\ \text{Diurnal effect index}\;\left(\text{DEI}\right)=\frac{\sqrt{{\left(\eta_{D}-\eta_{N}\right)}^{2}}}{\bar{\eta }} \end{array}$$ (6)

where η_H_, η_L_, η_D_, and η_N_ denote the parameter value during high tide, low tide, day, and night, respectively. $\bar{\eta }$ is the parameter mean for the entire measurement period.

To further assess whether surface water *P*co_2_ was driven by CO_2_-enriched groundwater inputs, we projected the linear regression of *P*co_2_ versus ^222^Rn to the expected groundwater end-member measurements (fig. S12). In this analysis, groundwater samples are expected to mix linearly with low *P*co_2_ and low ^222^Rn surface seawater. Any deviations from this simple mixing are interpreted as additional sources or sinks of CO_2_ (*90*). If the deviation is above the projected line (positive deviation), then additional CO_2_ sources are affecting observations. Contrastingly, if this deviation is below the projected line (negative deviation), then this can be attributed to separate CO_2_ sinks distinct from mixing. A deviation quotient (DQ) was calculated to quantify the magnitude of the deviation (Eq. 7)

$$\text{DQ}\;\left(\%\right)=\frac{D}{\bar{P_{{\text{CO}}_{2\text{gw}}}}}\times 100$$ (7)

where *D* is the distance of deviation from the linear projection (μatm) and $\bar{P_{{\text{CO}}_{2\text{gw}}}}$ is the mean of *P*co_2_ in groundwater end-member samples (μatm). Equation 8 was used to calculate the deviation from the projected line (*D*)

$$D=\left[\left(S\times \bar{{\text{Rn}}_{\text{gw}}}\right)+\text{In}\right]-\bar{P_{{\text{CO}}_{2\text{gw}}}}$$ (8)

where *S* represents the slope of correlation between surface water *P*co_2_ with ^222^Rn, *In* represent the intercept value between surface water *P*co_2_ and ^222^Rn, and $\bar{P_{{\text{CO}}_{2\text{gw}}}}$ is the mean of *P*co_2_ in groundwater end-member samples (μatm). The criteria that were chosen for acceptable range of deviation was −100% ≤ DQ ≤ 100%. If DQ was within this range, then it was inferred that surface water *P*co_2_ dynamics closely resemble that of the control and hence SGD is the primary driver of the CO_2_ dynamics in these coastal waters. The conclusions were drawn based on interpretation of the various lines of evidence mentioned.

## Supplementary Materials

### This PDF file includes:

Figs. S1 to S13 Tables S1 to S5 References
