# Supplementary material for: Groundwater releases CO2 to diverse global coastal ecosystems
Source: Sci Adv. 2025 Jan 10;11(2):eadr3240. doi: 10.1126/sciadv.adr3240 (PMC11721568; doi:10.1126/sciadv.adr3240)
Supplement: Supplementary file 1 — Figs. S1 to S13 Tables S1 to S5 References [file sciadv.adr3240_sm.pdf]

Science Advances
AAAS

Supplementary Materials for

Groundwater releases CO_2 to diverse global coastal ecosystems

Aprajita S. Tomer *et al.*

Corresponding author: Aprajita S. Tomer, aprajita.singh.tomer@gu.se

Sci. Adv. **11**, eadr3240 (2025)
DOI: 10.1126/sciadv.adr3240

This PDF file includes:

Figs. S1 to S13
Tables S1 to S5
References

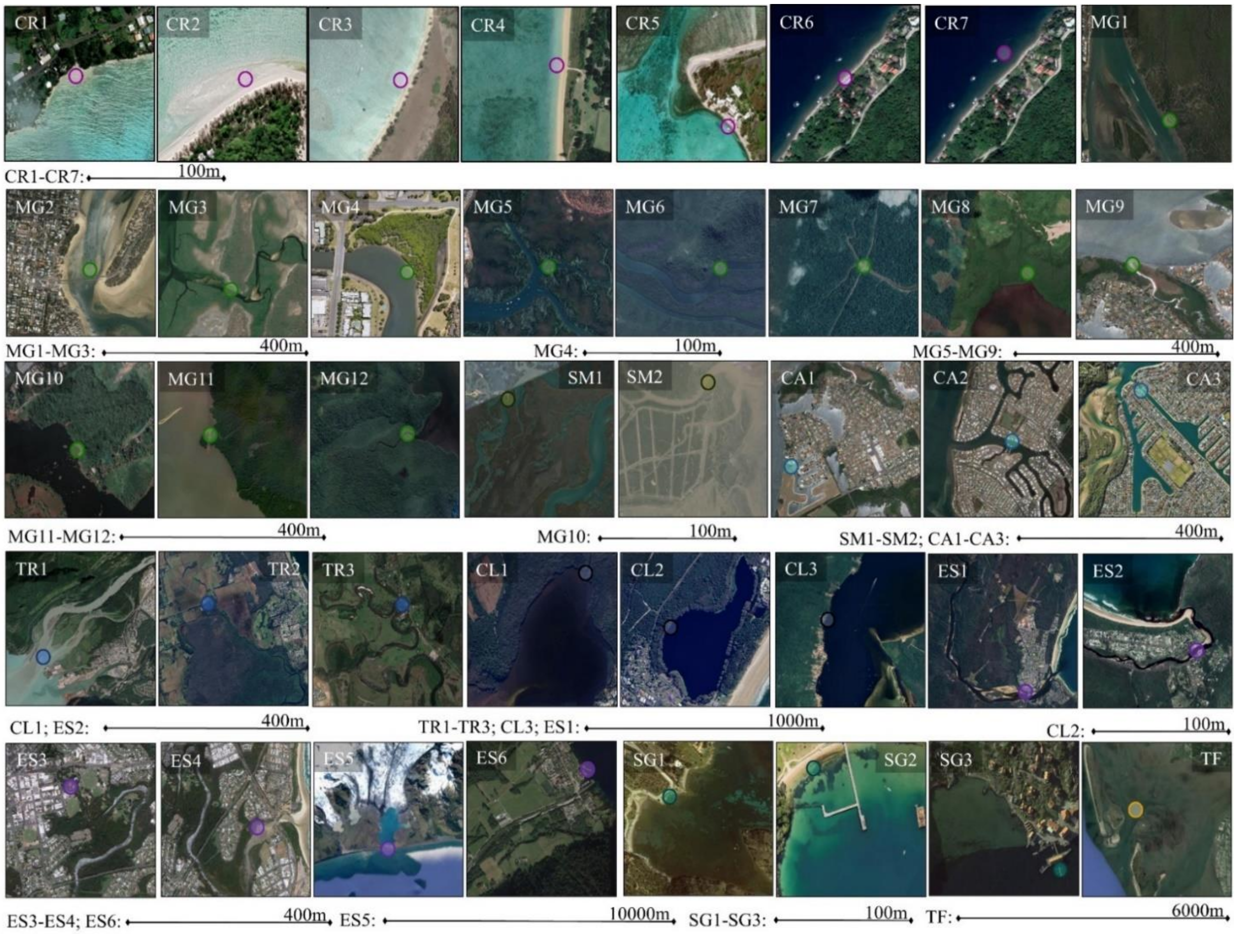

Fig. S1. Spatial images of individual study sites.

The image depicts the ecosystem type (images have been extracted from Google Earth). The location pin represents where surface water time series observations were made. Colour scheme of the location pins represents the ecosystem type as in Fig. 1.

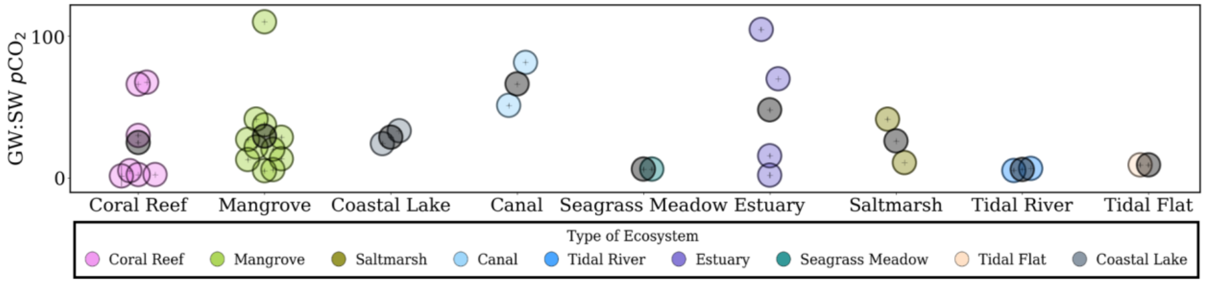

Fig. S2. CO_2 enrichment in groundwater.
Ratio of mean pCO_2 in groundwater to mean pCO_2 in surface water. The colour scheme represents ecosystem types. The black points represent the average for each ecosystem type.

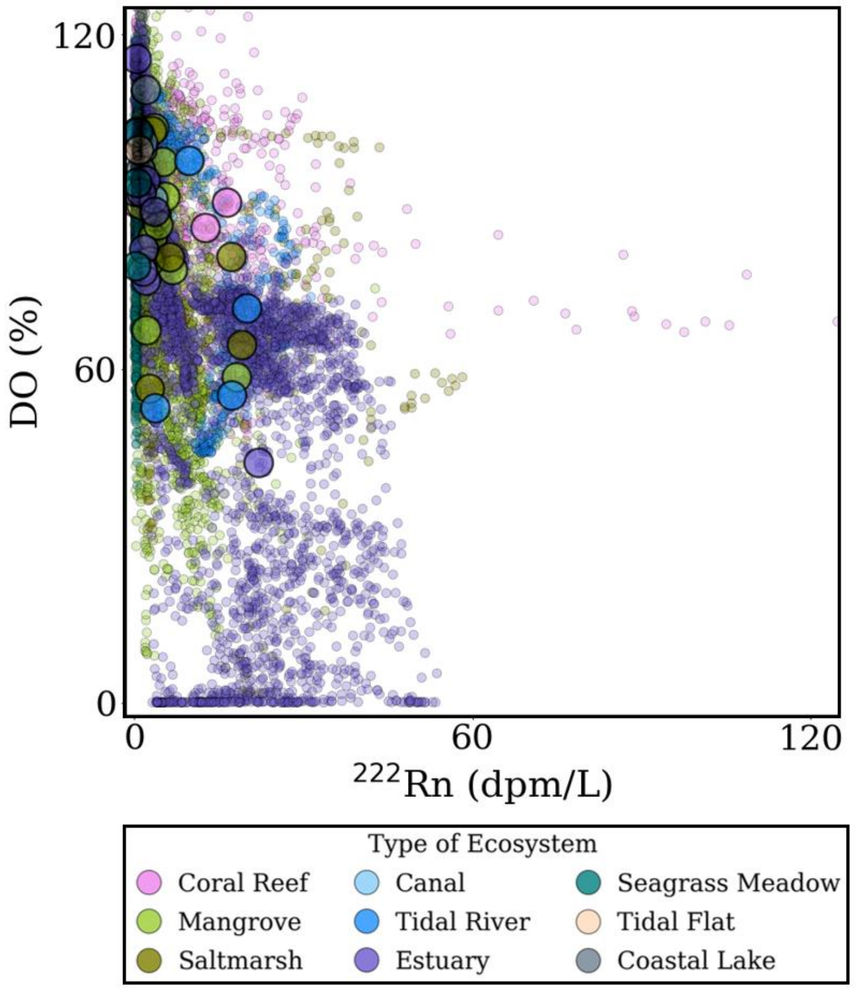

Fig. S3. Relationship between dissolved oxygen (DO) and ^{222}Rn.
The colour scheme represents ecosystem types. Smaller sized scatters represent individual data points and while bigger scatter points are representative of mean values of pCO_2 and ^{222}Rn in surface water of each site.

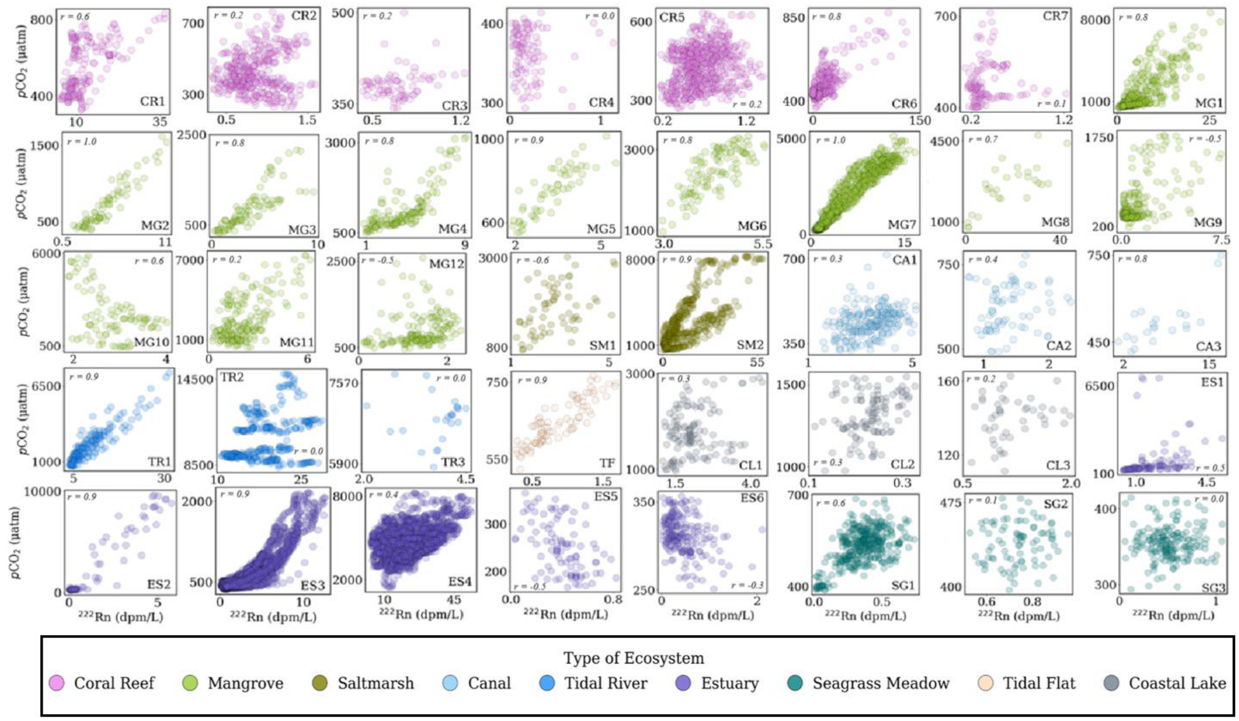

Fig. S4. Relationships between pCO$_2$ and ^{222}Rn at the 40 sites.

A positive correlation was observed in all 40 sites. r-values for each location have been mentioned in the individual plots. SM2 consists of data from SM2, SM2A, SM2B, and SM2C. CA2 consists of data from CA2, CA2A, and CA2B. TR2 consists of data from TR2 and TR2A. ES1 consists of data from ES1, ES1A, ES1B, and ES1C. The colour scheme represents ecosystem types.

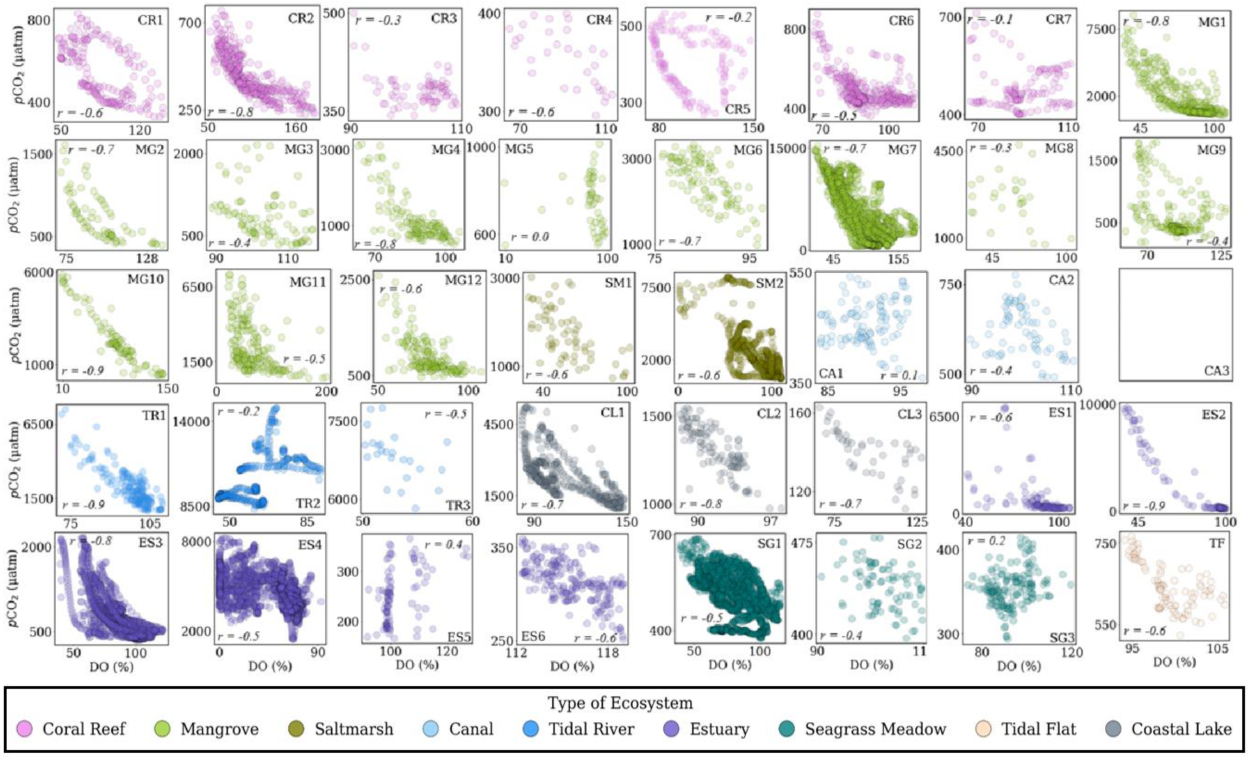

Fig. S5. Relationship between pCO_2 and dissolved oxygen (DO) at 40 sites.

A negative correlation was observed in all 40 sites. *r*-values for each location have been mentioned in the individual plots. SM2 consists of data from SM2, SM2A, SM2B, and SM2C. CA2 consists of data from CA2, CA2A, and CA2B. TR2 consists of data from TR2 and TR2A. ES1 consists of data from ES1, ES1A, ES1B, and ES1C. Correlations of pCO_2 with DO were stronger than with ^{222}Rn in only 9 out of 40 locations (see Figure 2b, Table S3). The colour scheme represents ecosystem type.

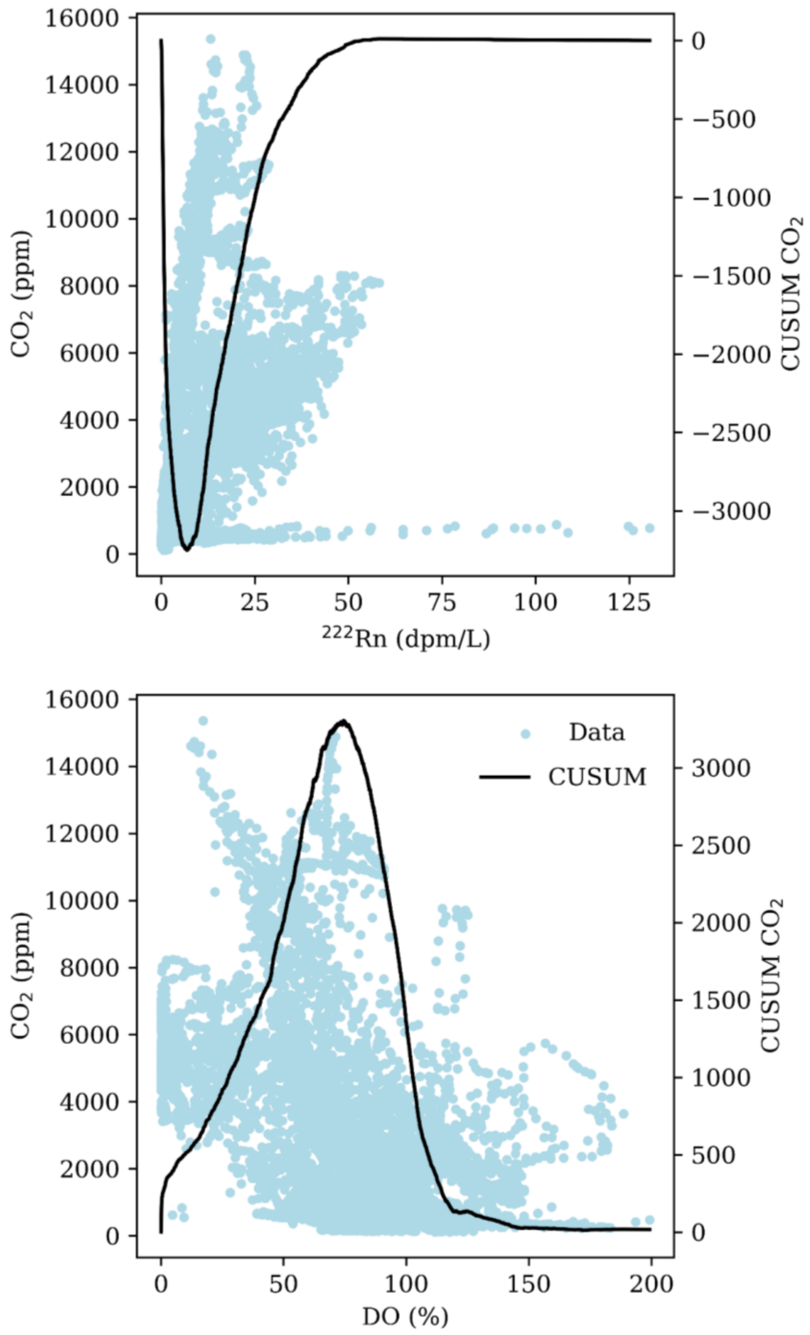

Fig. S6. CUSUMs driver-response relationships.

Plots show the driver-response relationships between the driving variables (^{222}Rn and DO) and response variable (CO_2). Underlying data (CO_2 vs ^{222}Rn, CO_2 vs DO) are shown in Figures S3 and S4. The CUSUM relationship for CO_2 (black line) shows the response of CO_2 to each driver. When ^{222}Rn > 5 dpm/L, increasing ^{222}Rn activities drive increasing CO_2 concentrations. Undersaturated DO values <75% drive increasing CO_2 concentrations, reflecting both biological activity and lower oxygen conditions typical of groundwater.

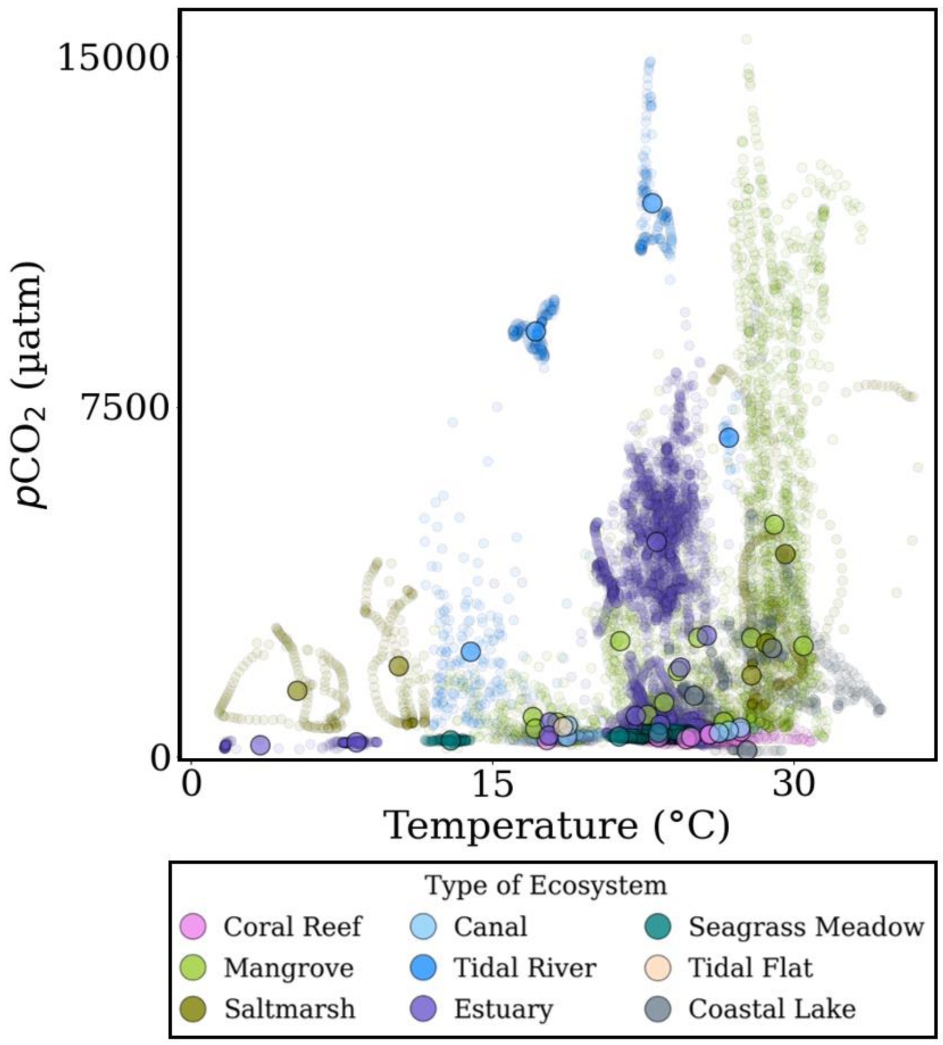

Fig. S7. Relationship between pCO_2 and Temperature.
Minimal to no relationship was observed in all 40 sites. The colour scheme represents ecosystem type**.** Smaller sized scatters represent individual data points and while bigger scatter points are representative of mean values of pCO_2 and temperature in surface water of each site.

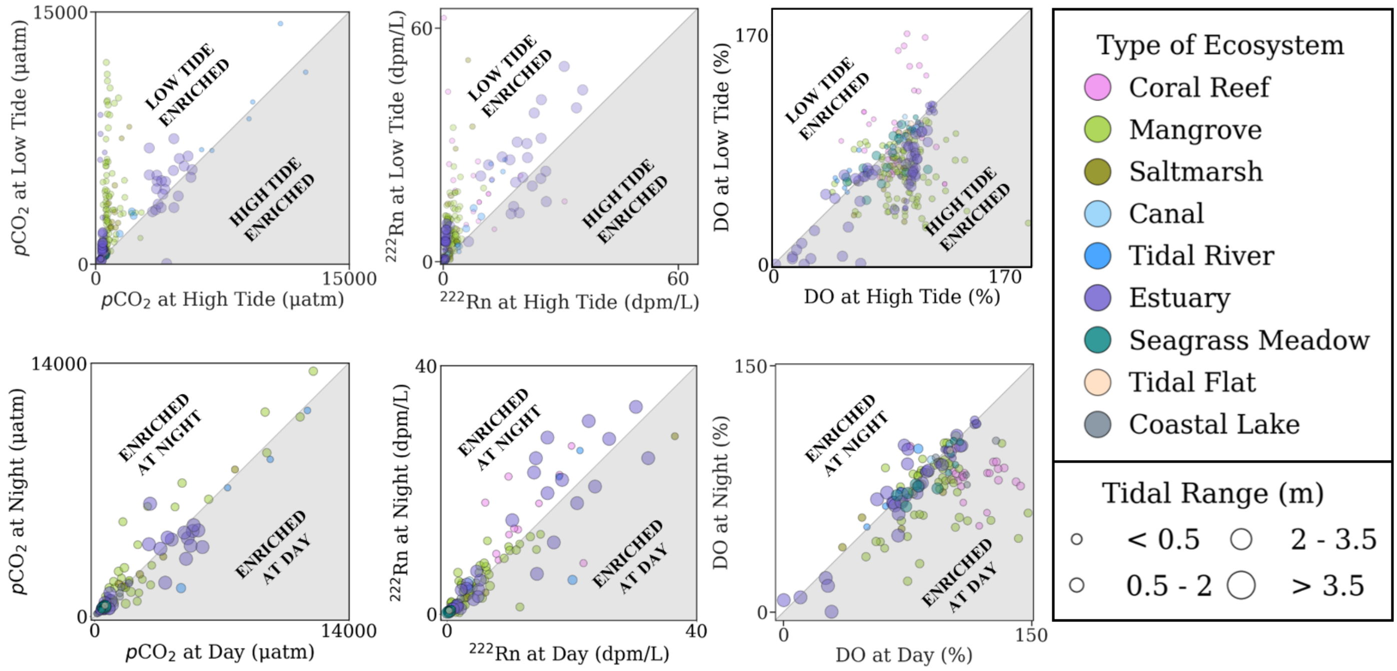

Fig. S8. Effect of tidal and diel cycles.

Comparative analysis of mean CO_2, ^{222}Rn, and DO levels during tidal and diel cycles. The symbol size represents tidal range observed in the 40 individual systems (m). The colour scheme represents different ecosystem types as in Figure 1. The white region in the top three plots represents the area where highest values were observed during low tide whereas the grey region represents the area where highest measurements were observed during high tide. The white region in the bottom three plots represents the area where highest values were observed during peak daylight hours whereas the grey region represents the area where highest values were observed during peak midnight hours.

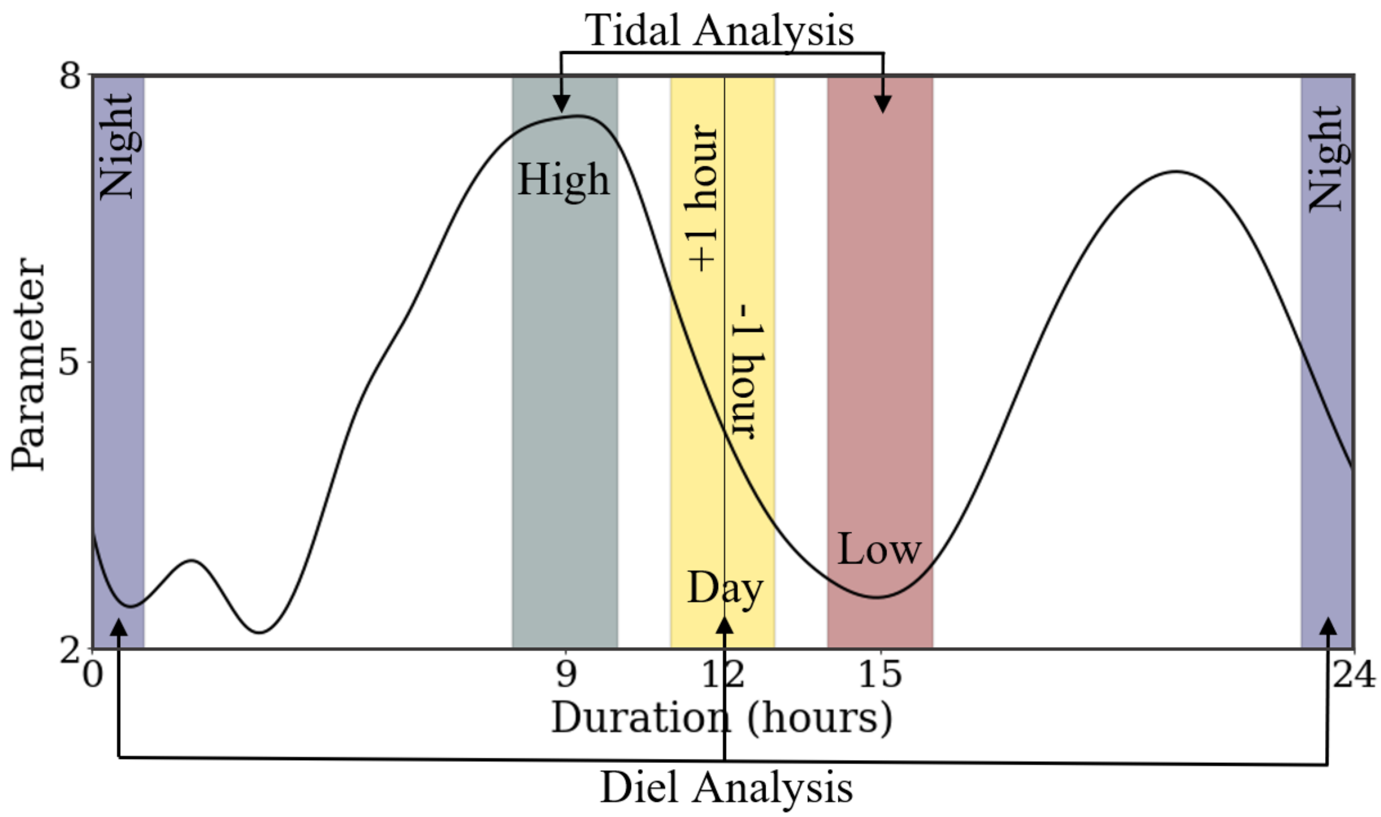

Fig. S9. Conceptual definition of the criteria used for tidal and diel analysis.
Tidal cycles capture the influence of SGD while diel oscillations capture the influence of biological productivity. Values of pCO_2, DO, and ^{222}Rn were calculated from the average during ±1 hour from 12:00 and 00:00 for the diel analysis (yellow and purple regions, respectively) and ±1 hour from high and low tides (green and red regions, respectively). The data represented here is part of time series observation from site MG5.

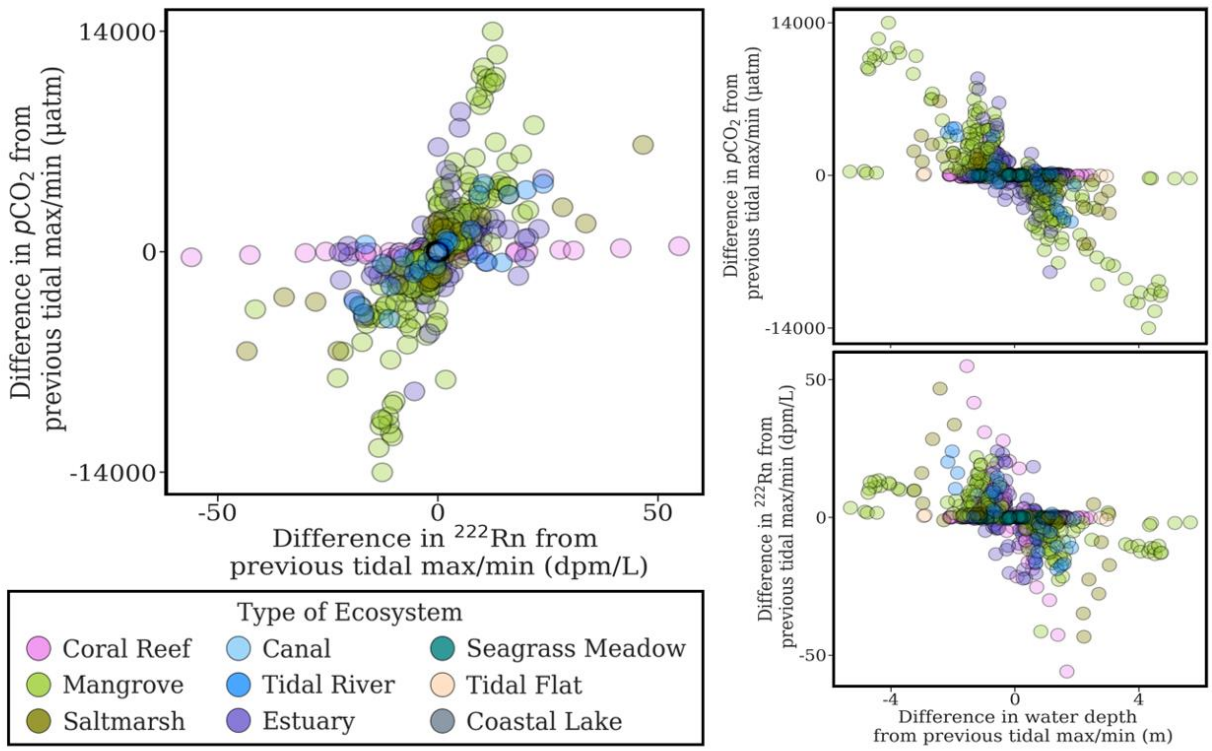

Fig. S10. The response of pCO_2 and ^{222}Rn to ebb and flood tides.
Negative values represent flood tides following maximum pCO_2 and ^{222}Rn often observed at low tide, and positive values represent ebb tides following minimum values often observed at high tide.

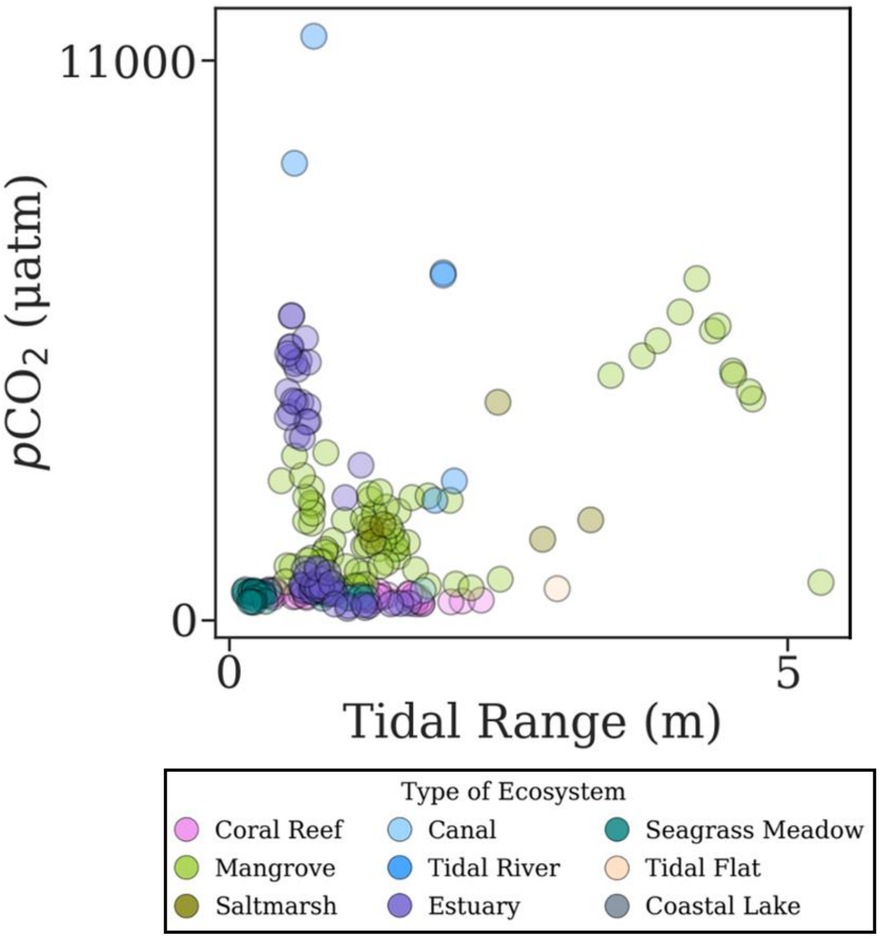

Fig. S11. Relationship between *p*CO_2 and tidal range.
Despite some scatter, an overall positive relationship was observed across all 40 sites implying that larger tides can enhance *p*CO_2. The colour scheme represents ecosystem types. Each dot represents a complete tidal cycle over about 12 hours.

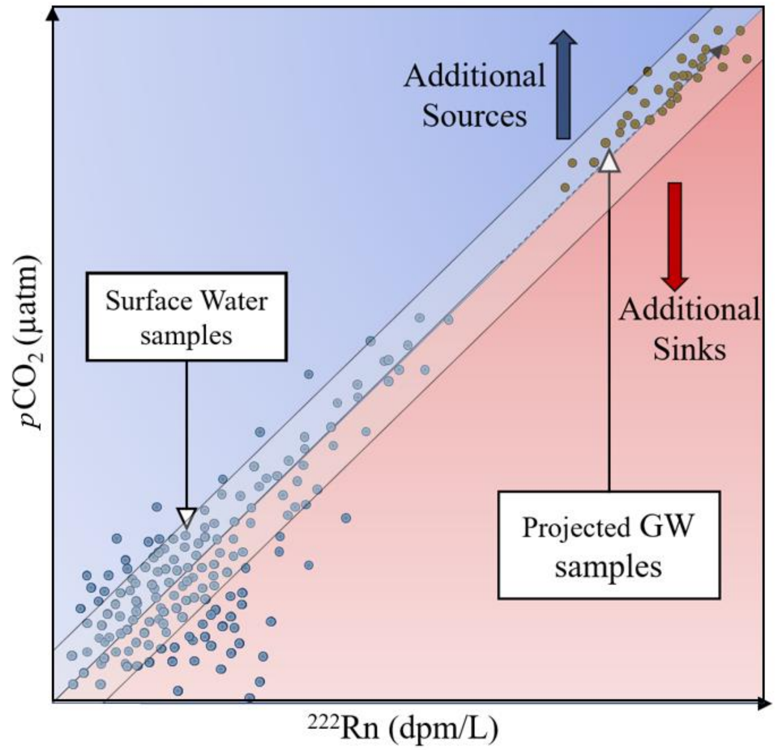

Fig. S12. Conceptual model representing calculation of deviation quotient from expected mixing between groundwater and seawater endmembers.

Brown scatter points represent the groundwater endmember sample, while blue scatter points represent surface water values. The area shaded in red represents the region depicting presence of other sources that remove CO_2 from the water column. The area shaded in blue represents the region depicting presence of other sources that add CO_2 to the water column. The region beyond the acceptable deviation limit on either side represents dominance of other CO_2 drivers. The data represented in the diagram are hypothetical.

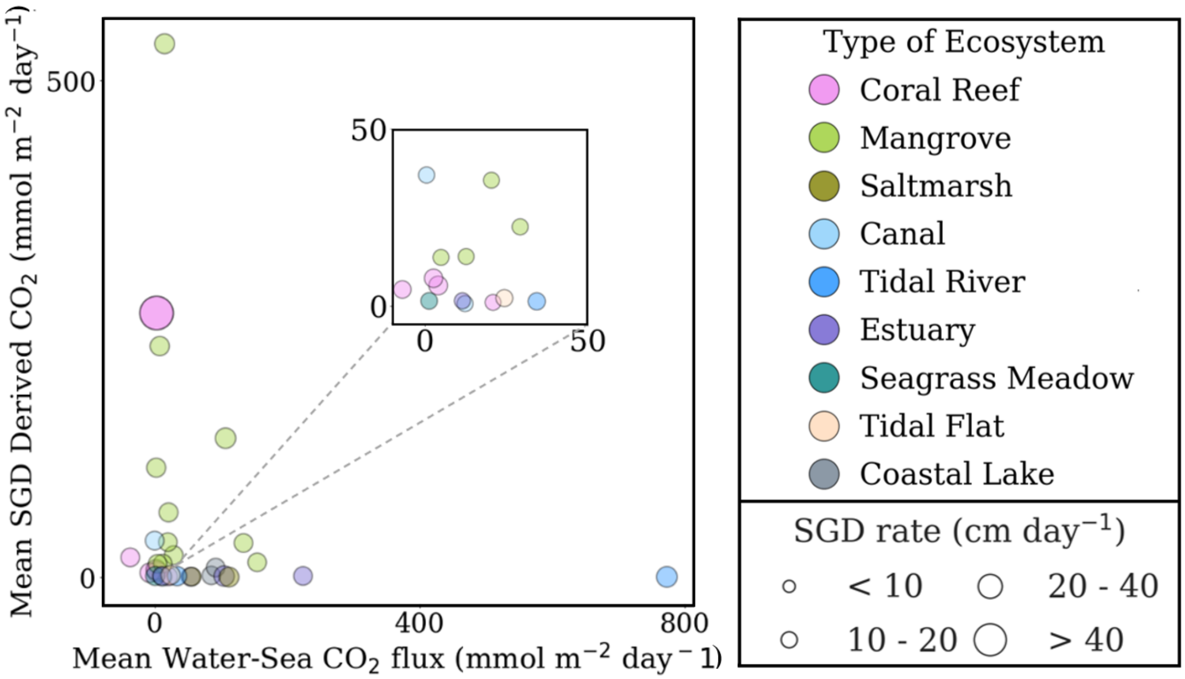

Fig. S13. SGD-derived CO_2 vs. water-air CO_2 fluxes.
The symbol size varies with SGD rate (cm/day) and the colour scheme represents ecosystem type.

Table S1. Study site details of the 40 sites and the sources of data.

Ecosystem	ID	Country	Coordinates	Source of Data	Sample size (days)
CORAL REEF	CR1	Cook Island	21.26°S; 159.74°W	(*91*)	3.77
	CR2	Australia	23.44°S; 151.92°E	(*92*)	9.36
	CR3	Australia	14.66°S; 145.45°E	(*93*)	1.82
	CR4	Australia	31.53°S; 159.07°E	(*93*)	4.75
	CR5	Australia	23.51°S; 152.09°E	(*94*)	13.08
	CR6	Philippines	13.52°N; 124.21°E	(*95*)	7.39
	CR7	Philippines	13.69°N; 123.89°E	(*95*)	2.54
MANGROVE	MG1	Australia	27.78°S; 153.38°E	(*52*)	15.89
	MG2	Australia	38.28°S; 144.49°E	(*52*)	5.5
	MG3	Australia	24.16°S; 151.89°E	(*52*)	5.13
	MG4	Australia	32.93°S; 151.78°E	(*52*)	5.83
	MG5	Australia	12.46°S; 130.84°E	(*52*)	4.88
	MG6	Australia	18.35°S; 146.30°E	(*52*)	6.13
	MG7	Brazil	0.87°S; 46.64°W	(*21*)	11.1
	MG8	Palau	7.37°N; 134.58°E	(*96*)	1.11
	MG9	Australia	29.42°S; 153.33°E	(*97*)	5.96
	MG10	India	9.92°N; 76.32°E	(*93*)	1
	MG11	Brazil	27.59°S; 48.56°W	(*51*)	6.56
	MG12	Brazil	23.22°S; 44.72°W	(*51*)	7.08
COASTAL LAKE	CL1	Australia	28.32°S; 153.56°E	(*31*)	3.02
	CL2	Australia	28.78°S; 153.58°E	(*98*)	2.75
	CL3	Brazil	27.54°S; 48.46°W	(*93*)	2
CANAL	CA1	Australia	29.42°S; 153.32°E	(*97*)	6.79
	CA2	Australia	27.05°S; 153.14°E	(*30*)	0.92
	CA2A				1.04
	CA2B				1.04
	CA3	Australia	28.10°S; 153.45°E	(*93*)	0.83
SEAGRASS MEADOW	SG1	Spain	42.29°N; 3.29°E	(*99*)	8.95
	SG2	Australia	33.84°S; 151.25°E	(*62*)	3.96
	SG3	Sweden	58.25°N, 11.44°E	(*100*)	3.67
ESTUARY	ES1	Australia	29.12°S; 153.43°E	(*50*)	1.21
	ES1A				1
	ES1B				1.08
	ES1C				1.04
	ES2	Australia	31.05°S; 153.06°E	(*101*)	2.06
	ES3	Australia	30.29°S; 153.12°E	(*32*)	16.03
	ES4	Australia	30.29°S; 153.14°E	(*32*)	19.79
	ES5	Iceland	64.04°N, 16.18°W	(*93*)	1.96
	ES6	Norway	62.21°N, 6.48°E	(*93*)	4.96
SALT MARSH	SM1	USA	33.35°N; 79.19°W	(*60*)	2.54
	SM2	China	33.03°N; 120.89°E	(*102*)	1
	SM2A				1
	SM2B				1
	SM2C				1
TIDAL RIVER	TR1	Canada	46.69°N; 123.18°W	(*103*)	4.7
	TR2	Australia	28.79°S; 153.56°E	(*104*)	1.2
	TR2A				1.09
	TR3	Australia	27.10°S; 153.01°E	(*29*)	1
TIDAL FLAT	TF	Germany	53.58°N; 6.95°E	(*63*)	1.16

Table S2: Summary values at each ecosystem type: Uncertainties for seagrass are missing due to absence of groundwater endmember data for these sites. The deviation quotient refers to the difference between pCO_2 projected at the observed ^{222}Rn groundwater endmember and the observed groundwater pCO_2 according to Figure S12.

					MEAN		MEDIAN			
**Ecosystem**	**CO_2 saturation (%)**	**pCO_2 (μatm)**	**DO (%)**	**^{222}Rn (dpm/L)**	**SGD-derived CO_2 flux (mmol/m^2/day)**	**Water-air CO_2 flux (mmol/m^2/day)**	**SGD-derived CO_2 flux (mmol/m^2/day)**	**Water-air CO_2 flux (mmol/m^2/day)**	**Deviation Quotient (%)**	**Tidal Effect/Diel Effect for pCO_2**
CORAL REEF	111 ± 17	446 ± 65	95 ± 7	5 ± 7	79 ± 126	-1 ± 18	6 ± 132	3 ± 15	74 ± 232	1.0
MANGROVE	450 ± 314	1816 ± 1271	83 ± 12	5 ± 5	103 ± 157	46 ± 54	36 ± 81	21 ± 45	-23 ± 60	3.4
COASTAL LAKE	316 ± 275	1260 ± 1095	99 ± 10	1 ± 1	5 ± 6	59 ± 53	5 ± 4	86 ± 70	372 ± 636	0.0
CANAL	142 ± 25	563 ± 91	98 ± 5	3 ± 3	52 ± 7	9 ± 5	53 ± 2	11 ± 8	-92 ± 6	1.8
SEAGRASS MEADOW	107 ± 21	439 ± 83	91 ± 12	1 ± 0	8	0 ± 4	5	1 ± 3	-73	0.7
ESTUARY	342 ± 350	1384 ± 1438	85 ± 20	4 ± 7	147 ± 41	61 ± 85	169 ± 141	12 ± 44	36 ± 322	5.9
SALTMARSH	577 ± 280	2377 ± 1159	77 ± 18	10 ± 8	191 ± 98	101 ± 60	226	73 ± 112	25 ± 84	10.8
TIDAL RIVER	1892 ± 1029	7512 ± 4062	69 ± 20	13 ± 7	726 ± 564	422 ± 724	1024 ± 512	73 ± 88	-57 ± 46	0.2
TIDAL FLAT	164 ± 14	644 ± 57	99 ± 3	1 ± 0	18	24 ± 17	8	24 ± 26	-68	5.8
TOTAL	455 ± 258	1826 ± 1036	88 ± 12	5 ± 4	148 ± 226	80 ± 133	36 ± 163	21 ± 62	-	-

Table S3: Groundwater observations: Mean and median values for all parameters at 40 nearshore sites.

Ecosystem	ID	Sample Size	MEAN Temperature (°C)	MEAN Salinity	MEAN DO (%)	MEAN pCO_2 (µatm)	MEAN ^{222}Rn (dpm/L)	MEDIAN Temperature (°C)	MEDIAN Salinity	MEDIAN DO (%)	MEDIAN pCO_2 (µatm)	MEDIAN ^{222}Rn (dpm/L)
CORAL REEF	CR1	3	28.0 ± 1.2	0.6 ± 0.3	49.0 ± 28.2	2873 ± 1017	359.0 ± 163.0	28.0	0.5	48.2	2392	206.0
	CR2	9	24.1 ± 0.8	19.3 ± 8.3	70.2 ± 21.5	656 ± 4864	65.0 ± 45.7	24.2	20.1	74.7	678	63.8
	CR3	1	28.3 ±0.4	0.1 ± 1.3	17.5 ± 4.0	952 ± 6064	59.0 ± 77.8	-	-	-	950	-
	CR4	9	17.8 ± 0.8	36.1 ± 13.2	44.7 ± 22.7	10915 ± 12050	48.0 ± 779.0	19.0	14.5	42.0	2774	20.4
	CR5	7	24.9 ± 1.5	35.7 ± 0.1	100.7 ± 20.3	1067 ± 76	15.0 ± 77.8	-	-	-	1059	-
	CR6	5	36.0 ± 4.0	21.0 ± 9.0	-	32420 ± 3275	232.0 ± 208.0	36.8	21.7	-	33946	165.0
	CR7	5	36.0 ± 4.0	21.0 ± 9.0	-	32420 ± 3275	232.0 ± 208.0	-	-	-	33946	-
MANGROVE	MG1	9	22.0 ± 6.2	28.5 ± 4.4	8.5 ± 6.3	24054 ± 10395	87.7 ± 36.1	26.3	28.2	4.9	24963	75.
	MG2	12	15.9 ± 0.7	35.3 ± 4.4	10.5 ± 13.7	16179 ± 7549	97.0 ± 49.4	15.5	35.3	4.1	12958	99.8
	MG3	12	26.7 ± 1.2	54.2 ± 17.0	4.0 ± 2.3	23310 ± 16345	97.7 ± 64.8	26.5	53.4	3.0	22623	85.1
	MG4	12	20.5 ± 0.8	35.1 ± 6.6	6.0 ± 3.5	48183 ± 15398	75.5 ± 46.8	20.4	36.6	5.3	51170	63.5
	MG5	12	28.3 ± 0.8	46.1 ± 6.6	12.9 ± 3.5	82717 ± 15398	24.6 ± 19.2	27.8	49.4	3.7	78196	23.0
	MG6	12	23.7 ± 4.1	40.1 ± 1.4	38.5 ± 34.1	12916 ± 11952	29.8 ± 34.7	21.1	40.4	22.0	7506	16.9
	MG7	10	29.5 ± 1.0	33.5 ± 2.5	33.0 ± 17.8	29749 ± 24083	44.3 ± 17.8	29.1	33.1	31.8	21804	43.0
	MG8	3	28.4 ± 1.0	9.6 ± 8.9	13.3 ± 8.8	34722 ± 7197	291.3 ± 77.8	28.3	5.6	5.7	32455	305.3
	MG9	11	14.2 ± 3.0	28.4 ± 10.0	64.3 ± 23.0	17433 ± 15971	21.1 ± 6.5	15.2	32.8	66.5	11476	21.4
	MG10	-	-	-	-	-	-	-	-	-	-	-
	MG11	12	22.7 ± 1.3	6.6 ± 7.1	-	52027 ± 21312	107.0 ± 208.6	23.1	31.5	-	42863	38.1
	MG12	12	23.1 ± 0.6	24.4 ± 2.7	-	33792 ± 77377	5.0 ± 2.3	23.1	25.0	-	12580	5.2
COASTAL LAKE	CL1	15	23.6 ± 2.0	0.5 ± 0.8	26.2 ± 20.6	56568 ± 72273	2526.0 ± 1982.0	23.7	0.1	23.5	32177	3027.0
	CL2	6	25.2 ± 0.5	0.1 ± 0.1	-	43764 ± 3414	8.9 ± 0.7	24.9	0.1	-	43024	8.7
	CL3	-	-	-	-	-	-	-	-	-	-	-
CANAL	CA1	6	16.9 ± 3.8	18.6 ± 11.8	67.5 ± 20.1	22351 ± 19248	14.4 ± 7.5	15.5	26.7	66.4	24351	19.0
	CA2						70.3 ± 63.3					
	CA2A	19	23.0 ± 1.7	1.9 ± 6.8	3.0 ± 4.7	50505 ± 23667	71.3 ± 64.3	22.9	0.2	-	51044	35.4
	CA2B						72.3 ± 65.3					
	CA3	-	-	-	-	-	-	-	-	-	-	-
SEAGRASS MEADOW	SG1	-	-	-	-	-	-	-	-	-	-	-
	SG2	10	22.5 ± 1.3	18.2 ± 13.0	-	2790 ± 3384	21.5 ± 20.3	22.5	21.8	-	1827	10.8
	SG3	-	-	-	-	-	-	-	-	-	-	-
ESTUARY	ES1, ES1A, ES1B, ES1C	6	20.5 ± 1.0	18.8 ± 8.1	28.0 ± 14.4	69798 ± 11054	32.5 ± 12.3	20.2	18.6	24.5	64604	32.4
	ES2	48	23.4 ± 3.2	15.1 ± 11.2	13.8 ± 17.6	5448 ± 9719	27.9 ± 23.4	24.6	19.4	9.3	58.0	25.0
	ES3	12	23.4 ± 3.1	18.8 ± 7.9	19.6 ± 23.8	72459 ± 101321	206.3 ± 848.7	23.2	17.6	8.0	23585	50.4
	ES4	12	23.4 ± 3.1	18.8 ± 7.9	19.6 ± 23.8	72459 ± 101321	206.3 ± 848.7	23.2	17.6	8.0	23585	50.4
	ES5	-	-	-	-	-	-	-	-	-	-	-
	ES6	-	-	-	-	-	-	-	-	-	-	-
SALT MARSH	SM1	12	28.6 ± 1.1	29.5 ± 9.8	-	36860 ± 31360	75.2 ± 44.8	28.9	34.0	-	7624	31.9
	SM2, SM2A, SM2B, SM2C	12	18.9 ± 1.3	22.9 ± 4.0	-	27734 ± 16	288.8 ± 137.3	-	-	-	28024	336.0
TIDAL RIVER	TR1	10	13.7 ± 0.8	3.9 ± 1.8	45.4 ± 33.2	12057 ± 15057	54.3 ± 26.5	13.6	3.3	26.9	4391	56.0
	TR2, TR2A	6	22.1 ± 2.2	0.2 ± 0.7	17.0 ± 11.1	71451 ± 28377	82.6 ± 64.6	22.6	0.1	15.9	71562	60.0
	TR3	12	25.0 ± 0.4	7.1 ± 8.2	19.3 ± 18.9	-	76.3 ± 49.3	25.0	3.5	15.3	-	60.1
TIDAL FLAT	TF	5	16.3 ± 0.6	31.1 ± 1.5	23.3 ± 13.7	6010 ± 8945	10.5 ± 14.9	16.0	31.4	25.1	1883	3.9

Table S4: Surface water observations: Mean and median values for all parameters at 40 nearshore sites. The R values represent correlations between pCO_2 and potential controls.

Ecosystem	ID	Sample Size	MEAN Temp-erature (°C)	MEAN Salinity	MEAN DO (%)	MEAN pCO_2 (µatm)	MEAN ^{222}Rn (dpm/L)	MEDIAN Temp-erature (°C)	MEDIAN Salinity	MEDIAN DO (%)	MEDIAN pCO_2 (µatm)	MEDIAN ^{222}Rn (dpm/L)	*r*-value ^{222}Rn	*r*-value DO	*r*-value Depth	*r*-value Sal-inity
	CR1	182	27.4 ± 1.8	36.1 ± 0.5	85.2 ± 23.5	549 ± 134	12.7 ± 5.9	27.3 ± 2.4	36.1 ± 0.8	81.4 ± 34.6	531 ± 237	10.6 ± 5.2	0.6	-0.6	-0.5	-0.6
	CR2	401	23.3 ± 1.4	37.1 ± 0.1	101.7 ±29.8	425 ± 109	0.9 ± 0.3	22.9 ± 1.4	37.2 ± 0.2	93.1 ± 39.9	399 ± 159	0.8 ± 0.4	0.2	-0.8	-0.1	0.5
	CR3	74	24.6 ± 0.5	35.8 ± 0.1	102.4 ± 4.7	382 ± 24	0.7 ± 0.2	24.8 ± 0.5	35.8 ± 0.2	104.1 ± 7.6	381 ± 25	0.7 ± 0.2	0.2	-0.3	0.0	-0.1
CORAL REEF	CR4	105	17.8 ± 0.6	36.1 ± 0.3	91.9 ± 14.9	363 ± 29	0.2 ± 0.2	17.8 ± 0.7	36.2 ± 0.2	92.4 ± 23.8	371 ± 47	0.2 ± 0.2	-0.0	-0.6	-0.2	-0.1
	CR5	619	24.9 ± 1.5	35.7 ± 0.1	100.7 ±20.3	429 ± 76	0.7 ± 0.2	24.7 ± 2.4	35.7 ± 0.1	96.7 ± 38.4	430 ± 119	0.6 ± 0.3	0.2	-0.2	0.0	-0.4
	CR6	355	25.9 ± 0.3	33.6 ± 0.5	89.8 ± 10.6	490 ± 82	16.5 ±20.5	25.8 ± 0.4	33.6 ± 0.8	87.8 ± 16.0	460 ± 76	10.8 ±16.7	0.8	-0.5	-0.6	-0.4
	CR7	123	25.8 ± 0.3	33.7 ± 0.5	89.8 ± 11.8	480 ± 66	0.4 ± 0.2	25.7 ± 0.5	33.6 ± 0.8	88.2 ± 16.1	464 ± 86	0.3 ± 0.2	0.1	-0.1	-0.4	-0.0
	MG1	390	24.2 ± 2.3	35.9 ± 3.6	82.0 ± 18.4	1005 ± 1642	5.2 ± 5.1	24.3 ± 2.4	36.5 ± 1.3	82.0 ± 28.6	1005 ± 1968	5.1 ± 6.3	0.8	-0.8	-0.8	0.1
	MG2	72	18.2 ± 1.4	34.6 ± 1.4	96.8 ± 16.3	648 ± 298	4.6 ± 2.3	18.1 ± 1.7	34.9 ± 1.2	96.8 ± 20.1	648 ± 387	3.8 ± 2.9	1.0	-0.7	-0.7	-0.7
	MG3	93	17.0 ± 1.5	37.7 ± 1.1	102.9 ± 7.5	734 ± 440	3.2 ± 2.1	16.8 ± 2.7	37.8 ± 1.6	102.9 ±11.7	734 ± 503	3.0 ± 3.0	0.8	-0.4	-0.7	0.7
	MG4	140	23.6 ± 0.8	31.8 ± 1.5	88.1 ± 9.9	942 ± 617	3.8 ± 1.9	23.6 ± 1.2	31.4 ± 2.4	88.2 ± 14.5	942 ± 565	3.8 ± 3.0	0.8	-0.8	-0.8	-0.4
	MG5	53	26.5 ± 0.4	34.1 ± 2.7	87.2 ± 17.9	762 ± 116	3.3 ± 0.9	26.5 ± 0.6	35.1 ± 0.4	87.3 ± 7.2	762 ± 200	3.4 ± 1.2	0.9	-0.0	-0.8	0.1
MANGROVE	MG6	104	21.4 ± 0.6	35.3 ± 0.1	85.1 ± 5.1	2526 ± 545	4.3 ± 0.6	21.2 ± 0.8	35.3 ± 0.1	87.2 ± 7.8	2526 ± 881	4.0 ± 0.9	0.8	-0.7	-1.0	-0.7
	MG7	1525	29.0 ± 1.0	34.6 ± 1.0	95.1 ± 33.7	3814 ± 3590	4.3 ± 4.2	28.8± 1.6	34.4 ± 1.0	94.8 ± 48.9	3753 ± 5650	4.1 ± 7.7	1.0	-0.7	-0.9	0.9
	MG8	27	27.9 ± 0.7	15.9 ± 4.4	62.4 ± 18.2	2633 ± 997	15.4 ±11.6	27.9 ± 1.2	16.4 ± 7.0	62.4 ± 21.5	2809 ± 1060	26.4 ±18.2	0.7	-0.3	-0.8	-0.4
	MG9	230	17.1 ± 2.3	33.5 ± 3.2	91.5 ± 14.8	434 ± 374	0.7 ± 1.4	17.8 ± 2.7	33.3 ± 4.7	90.0 ± 13.2	434 ± 298	0.7 ± 1.1	-0.5	-0.4	-0.5	-0.2
	MG10	94	30.5 ± 0.9	17.5 ± 0.2	81.6 ± 34.4	2087 ± 1469	2.8 ± 0.5	30.8 ± 0.8	17.6 ± 0.2	81.5 ± 35.6	2087 ± 1449	2.8 ± 0.8	0.6	-0.9	0.1	-0.1
	MG11	182	25.2 ± 2.1	31.6 ± 1.3	57.4 ± 34.6	1955 ± 1667	1.9 ± 1.3	25.6 ± 3.0	32.1 ± 2.3	57.4 ± 40.1	1954 ± 2402	1.9 ± 1.5	0.2	-0.5	-0.7	0.2
	MG12	169	22.7 ± 0.5	29.1 ± 3.2	78.0 ± 10.6	750 ± 382	1.4 ± 0.5	22.8 ± 0.6	25.6 ± 5.0	78.1 ± 14.4	750 ± 404	1.4 ± 0.6	-0.5	-0.6	-0.4	-0.4
	CL1	426	28.9 ± 2.5	21.6 ± 0.4	110.0 ±20.8	2328 ± 975	2.1 ± 0.7	28.7 ± 4.0	21.5 ± 0.5	102.0 ±39.0	2167 ± 1264	1.9 ± 0.9	0.3	-0.7	0.6	0.5
COASTAL LAKE	CL2	111	25.0 ± 0.5	0.1 ± 0.0	91.7 ± 2.1	1312 ± 134	0.2 ± 0.0	24.9 ± 0.9	0.1 ± 0.0	91.5 ± 3,7	1321 ± 191	0.2 ± 0.0	0.3	-0.8	-0.4	-
	CL3	45	27.7 ± 0.8	24.1 ± 0.1	95.6 ± 18.6	140 ± 12	1.2 ± 0.4	27.6 ± 1.2	24.1 ± 0.2	91.5 ± 34.4	142 ± 14	1.1 ± 0.4	0.2	-0.7	0.3	0.3
	CA1	274	18.7 ± 0.9	31.6 ± 1.9	90.4 ± 3.4	436 ± 56	3.4 ± 0.8	18.8 ± 1.2	31.3 ± 2.9	90.5 ± 4.7	434 ± 70	3.4 ± 1.2	0.3	0.1	0.0	-0.1
	CA2	21	27.3 ± 0.6	28.6 ± 0.2	101.8 ± 5.2	625 ± 64	1.1 ± 0.2	27.4 ± 1.1	28.7 ± 0.4	102.8 ± 8.8	615 ± 132	1.1 ± 0.2	0.0	-0.2	-0.6	-0.4
CANAL	CA2A	26	18.8 ± 0.2	33.8 ± 0.2	99.3 ± 2.0	670 ± 46	1.4 ± 0.3	18.8 ± 0.4	33.8 ± 0.1	99.3 ± 3.3	673 ± 53	1.4 ± 0.4	0.5	-0.5	-0.7	0.0
	CA2B	24	26.8 ± 0.4	35.3 ± 0.1	100.5 ± 5.9	565 ± 46	1.6 ± 0.4	26.8 ± 0.4	35.3 ± 0.2	101.8 ± 8.7	564 ± 59	1.5 ± 0.6	0.7	-0.6	-0.5	-0.5
	CA3	21	26.3 ± 0.6	17.5 ± 2.7	-	518 ± 82	7.7 ± 4.5	26.3 ± 0.7	16.2 ± 3.9	-	504 ± 74	6.6 ± 7.7	0.8		-0.4	0.0
	SG1	2398	23.2 ± 0.7	36.6 ± 0.9	78.4 ± 15.9	522 ± 64	0.4 ± 0.2	23.2 ± 0.9	36.6 ± 1.2	76.5 ± 24.1	532 ± 86	0.4 ± 0.2	0.6	-0.5	-0.2	0.4
SEAGRASS MEADOW	SG2	86	21.3 ± 0.4	36.5 ± 0.6	102.4 ± 4.3	439 ± 19	0.7 ± 0.1	21.3 ± 0.6	36.4 ± 1.0	101.9 ± 5.5	441 ± 28	0.7 ± 0.1	0.1	-0.4	0.0	0.1
	SG3	177	12.9 ± 0.6	21.0 ± 1.1	93.3 ± 8.2	355 ± 22	0.5 ± 0.2	12.9 ± 0.8	20.8 ± 1.1	92.7 ± 13.2	354 ± 24	0.5 ± 0.3	0.0	0.2	0.0	0.0
	ES1	29	17.8 ± 0.9	29.6 ± 7.9	93.8 ± 7.8	741 ± 851	2.0 ± 1.1	18.0 ± 0.9	33.8 ±10.1	93.9 ± 13.1	465 ± 192	2.2 ± 2.1	0.5	-0.6	-0.6	-0.9
	ES1A	25	24.4 ± 1.4	20.0±12.2	81.4 ± 11.3	1913 ± 2247	1.8 ± 0.6	24.8 ± 2.4	27.2 ±20.9	83.5 ± 20.2	826 ± 1263	1.6 ± 0.9	-0.0	-0.7	-0.3	-0.8
	ES1B	22	17.8 ± 1.5	31.7 ± 3.2	91.5 ± 5.6	459 ± 110	1.3 ± 0.8	17.5 ± 2.3	33.2 ± 4.9	90.8 ± 7.2	423 ± 185	1.2 ± 0.8	0.7	-0.3	-0.7	-0.9
	ES1C	26	22.1 ± 2.1	35.2 ± 0.6	75.8 ± 21.3	879 ± 627	2.1 ± 1.3	21.9 ± 2.4	35.3 ± 0.9	80.4 ± 38.6	631 ± 642	1.9 ± 2.0	0.8	-0.7	-0.7	-0.7
ESTUARY	ES2	85	25.7 ± 0.9	25.4 ±10.9	77.4 ± 23.2	2600 ± 3252	1.6 ± 1.8	25.4 ± 0.7	33.6 ±21.1	91.9 ± 44.9	374 ± 4720	0.4 ± 2.9	0.9	-0.9	-0.8	-0.9
	ES3	2162	23.4 ± 1.1	24.1 ± 6.2	88.1 ± 16.1	692 ± 357	3.7 ± 2.7	23.4 ± 1.4	25.8 ± 9.2	90.3 ± 24.4	567 ± 387	3.3 ± 3.9	0.9	-0.8	-0.6	-0.7
	ES4	2658	23.2 ± 1.2	8.9 ± 10.8	43.1 ± 26.6	4604 ± 1062	22.1 ± 9.6	23.4 ± 1.6	2.7 ± 18.4	56.1 ± 48.4	4519 ± 1538	20.9 ±12.6	0.4	-0.5	-0.3	0.4
	ES5	95	3.4 ± 2.6	15.8 ± 9.2	102.4 ± 6.8	252 ± 55	0.4 ± 0.2	1.7 ± 5.1	9.8 ± 18.4	99.4 ± 9.2	248 ± 98	0.4 ± 0.2	-0.5	0.4	0.4	0.4
	ES6	239	8.2 ± 0.4	28.1 ± 0.6	115.6 ± 1.8	314 ± 22	0.4 ± 0.3	8.3 ± 0.4	28.1 ± 0.5	115.3 ± 2.8	312 ± 29	0.4 ± 0.4	-0.3	-0.6	0.0	-0.1
	SM1	62	27.9 ± 1.5	21.5 ± 8.6	56.4 ± 18.4	1747 ± 592	2.8 ± 0.9	28.5 ± 2.2	22.3 ±14.1	53.9 ± 19.0	1680 ± 845	2.7 ± 1.4	0.5	-0.6	-0.7	-0.7
	SM2	145	29.6 ± 2.5	15.7 ± 3.3	64.2 ± 26.4	4343 ± 2537	19.1 ±15.2	29.1 ± 2.9	16.4 ± 6.0	71.4 ± 27.3	3515 ± 5207	16.2 ±21.7	0.8	-0.7	-0.9	-0.6
SALTMARSH	SM2A	145	28.6 ± 0.9	21.4 ± 3.1	79.9 ± 12.9	2433 ± 1187	6.4 ± 4.5	28.3 ± 1.3	23.3 ± 5.3	79.5 ± 25.9	2131 ± 1993	5.9 ± 7.1	0.9	-0.8	-0.9	-0.8
	SM2B	145	10.3 ± 0.9	21.1 ± 2.4	80.0 ± 16.4	1943 ± 1152	17.3 ±12.0	10.6 ± 1.4	21.1 ± 3.8	81.6 ± 31.4	1670 ± 2281	16.9 ±21.8	0.9	-0.2	-0.8	-0.1
	SM2C	145	5.3 ± 1.9	22.9 ± 0.6	102.6 ± 5.1	1418 ± 667	3.2 ± 3.3	5.8 ± 2.7	22.8 ± 0.4	104.4 ± 8.2	1295 ± 1174	1.8 ± 3.2	0.3	-0.6	-0.4	0.5
	TR1	174	13.9 ± 1.5	2.0 ± 0.7	97.3 ± 8.0	2252 ± 1278	9.8 ± 5.6	13.8 ± 2.2	1.8 ± 0.6	99.7 ± 7.8	2000 ± 1542	7.9 ± 6.3	0.9	-0.9	-0.9	0.2
TIDAL RIVER	TR2	174	17.2 ± 0.6	0.2 ± 0.0	55.2 ± 7.5	9109 ± 332	17.4 ± 4.4	17.3 ± 0.9	0.2 ± 0.1	55.4 ± 14.7	9097 ± 480	17.0 ± 7.2	-0.2	-0.3	0.0	0.2
	TR2A	151	22.9 ± 0.5	0.2 ± 0.0	70.8 ± 9.9	11852 ±1096	20.0 ± 4.1	22.7 ± 0.9	0.2 ± 0.1	69.8 ± 13.8	11524±1210	19.3 ± 6.6	0.2	-0.1	-0.4	-0.4
	TR3	24	26.8 ± 0.2	0.8 ± 1.2	52.9 ± 2.2	6834 ± 472	3.8 ± 0.6	26.8 ± 0.3	0.2 ± 0.4	52.4 ± 2.7	6882 ± 483	3.9 ± 0.5	0.0	-0.5	-0.4	-0.5
TIDAL FLAT	TF	94	18.5 ± 0.2	28.9 ± 0.0	99.2 ± 2.9	644 ± 57	0.8 ± 0.4	18.5 ± 0.2	28.9 ± 0.0	98.7 ± 3.8	636 ± 93	0.7 ± 0.6	0.9	-0.6	-0.7	0.0

Table S5. Summary information on CO_2 fluxes: SGD rates and fluxes along with CO_2 efflux rates at 40 nearshore sites. Uncertainties calculated here refer to standard errors.

Ecosystem	ID	Tidal Range (m)	SGD rate (cm/day)	Mean SGD-derived CO_2 Flux (mmol/m^2/day)	Median SGD-derived CO_2 Flux (mmol/m^2/day)	Mean Water-air CO_2 Flux (mmol/m^2/day)	Median Water-air CO_2 Flux (mmol/m^2/day)
CORAL REEF	CR1	0.6	1.1 ± 0.8	1.0 ± 0.8	0.8	5.8 ± 701.1	3.8
	CR2	1.8	29.7 ± 10.3	5.8 ± 8.4	5.8	4.6 ± 16.3	0.5
	CR3	2.4	17.4 ± 11.9	4.6 ± 0.7	4.5	-6.9 ± 7.9	-7.4
	CR4	2.3	4.8 ± 5.6	20.1 ± 1.6	4.7	-36.3 ± 40.4	-19.2
	CR5	1.5	25.8 ± 20.4	7.8 ± 0.8	7.7	2.7 ± 11.3	3.2
	CR6	1.7	35 ± 48	266.0	266.0	-0.8 ± 18.1	1.5
	CR7	1.7	35 ± 48	266.0	266.0	3.4 ± 3.7	2.2
MANGROVE	MG1	1.8	8.4 ± 1.8	65.4 ± 0.2	60.8	21.5 ± 61.3	4.6
	MG2	1.4	3.7 ± 2.4	22.3 ± 0.8	18.3	29.2 ± 41.5	6.6
	MG3	2.6	2.1 ± 1.4	13.9 ± 1.0	13.9	12.7 ± 16.8	4.9
	MG4	1.2	14.7 ± 1.4	232.7 ± 0.3	238.4	8.2 ± 15.8	2.6
	MG5	5.7	24.9 ± 1.4	537.0 ± 0.2	517.2	15.5 ±20.2	5.5
	MG6	2.3	35.5 ± 1.4	140.0 ± 0.9	84.7	107.5 ± 65.0	100.9
	MG7	4.0	1.8 ± 1.6	15.2 ± 1.2	12.9	152.1 ± 161.2	110.9
	MG8	1.6	3.3 ± 3.3	34.6 ± 1.0	31.6	134.5 ± 79.5	122.3
	MG9	1.0	2 ± 1.1	13.7 ± 1.1	8.7	5.0 ± 11.6	0.4
	MG10	0.5	-	-	-	45.1 ± 53.1	21.7
	MG11	1.0	1.8 ± 1.6	35.5 ± 1.0	35.5	20.4 ± 26.6	10.3
	MG12	1.6	10.7 ± 3.9	110.4 ± 2.3	110.4	2.9 ± 5.5	1.3
COASTAL LAKE	CL1	0.2	0.1 ± 1.4	2.0 ± 14.0	1.1	85.9 ± 84.3	62.0
	CL2	0.1	0.7 ± 1.4	9.9 ± 2.1	9.8	92.6 ± 68.0	80.6
	CL3	-	-	-	-	-2.2 ± 2.4	-1.4
CANAL	CA1	4.0	4.4 ± 1.4	37.0 ± 0.9	46.3	112.7 ± 107.1	70.6
	CA2	1.4				13.7 ± 9.3	8.7
	CA2A	1.7	2.9 ± 1.3	51.8 ± 0.6	53.3	12.6 ± 6.6	10.7
	CA2B	1.8				10.7 ± 7.7	8.8
	CA3	1.7	-	-	-	5.9 ± 6.0	3.9
SEAGRASS MEADOW	SG1	0.5	-	-	-	3.7 ± 5.6	1.9
	SG2	1.2	8.7 ± 5.8	8.1 ± 1.4	5.3	1.3 ± 1.9	0.6
	SG3	0.4	-	-	-	-3.6 ± 4.6	-2.2
ESTUARY	ES1	0.8				11.8 ± 30.3	1.7
	ES1A	0.7	7.3 ± 4.8	180.3 ± 0.7	168.9	171.7 ± 345.2	19.2
	ES1B	1.2				6.4 ± 10.3	2.8
	ES1C	1.2				30.1 ± 46.4	10.9
	ES2	1.2	35 ± 12	106.1 ± 1.3	31.1	105.4 ± 307.1	0.0
	ES3	1.2	3.7 ± 2.8	102.5 ± 1.4	24.2	11.7 ± 16.8	5.1
	ES4	0.8	3.7 ± 2.8	102.5 ± 1.4	24.2	224.3 ± 222.5	145.7
	ES5	1.4	-	-	-	-7.2 ± 9.9	-3.7
	ES6	1.6	-	-	-	-2.2 ± 3.3	-1.2
SALT MARSH	SM1	1.4	1.6 ± 1.1	16.1 ± 0.7	3.2	56.4 ± 39.9	49.4
	SM2	3.5				72.6 ± 46.3	54.6
	SM2A	2.4	23.3 ± 7.2	226.5 ± 0.3	223.5	197.9 ± 123.7	199.6
	SM2B	2.7				122.8 ± 122.8	58.8
	SM2C	2.7				57.3 ± 38.0	49.8
TIDAL RIVER	TR1	2.3	14.9 ± 3.1	74.3 ± 1.3	26.8	34.3 ± 62.7	7.1
	TR2	0.8	38.9 ± 12.2	1037.9 ± 0.5	1024.4	39.3 ± 38.3	43.5
	TR2A	0.8	39.9 ± 12.2	1064.6 ± 0.5	1050.8	1507.7 ± 145.1	1461.1
	TR3	1.9	26.8 ± 1.4	-	-	107.1 ± 77.7	91.9
TIDAL FLAT	TF	3.1	11.8 ± 1,4	17.6 ± 2.2	8.1	24.4 ± 17.0	18.3

REFERENCES AND NOTES

1. P. Regnier, L. Resplandy, R. G. Najjar, P. Ciais, The land-to-ocean loops of the global carbon cycle. *Nature* **603**, 401–410 (2022).

2. W.-J. Cai, Estuarine and coastal ocean carbon paradox: CO_2 sinks or sites of terrestrial carbon incineration? *Ann. Rev. Mar. Sci.* **3**, 123, 145 (2011).

3. J. A. Rosentreter, G. G. Laruelle, H. W. Bange, T. S. Bianchi, J. J. M. Busecke, W.-J. Cai, B. D. Eyre, I. Forbrich, E. Y. Kwon, T. Maavara, N. Moosdorf, R. G. Najjar, V. V. S. S. Sarma, B. Van Dam, P. Regnier, Coastal vegetation and estuaries are collectively a greenhouse gas sink. *Nat. Clim. Chang.* **13**, 579–587 (2023).

4. A. V. Borges, G. Abril Carbon, Dioxide and Methane Dynamics in Estuaries In Treatise on Estuarine and Coastal Science. W. Eric M. Donald Eds (Academic Press, 2011).

5. C.-T. A. Chen, T.-H. Huang, Y.-C. Chen, Y. Bai, X. He, Y. Kang, Air-sea exchanges of CO_2 in the world's coastal seas. *Biogeosciences* **10**, 6509–6544 (2013).

6. F. Lacroix, T. Ilyina, J. Hartmann, Oceanic CO_2 outgassing and biological production hotspots induced by pre-industrial river loads of nutrients and carbon in a global modeling approach. *Biogeosciences* **17**, 55–88 (2020).

7. G. M. S. Reithmaier, A. Cabral, A. Akhand, M. J. Bogard, A. V. Borges, S. Bouillon, D. J. Burdige, M. Call, N. Chen, X. Chen, L. C. Cotovicz Jr., M. J. Eagle, E. Kristensen, K. D. Kroeger, Z. Lu, D. T. Maher, J. L. Pérez-Lloréns, R. Ray, P. Taillardat, J. J. Tamborski, R. C. Upstill-Goddard, F. Wang, Z. A. Wang, K. Xiao, Y. Y. Y. Yau, I. R. Santos, Carbonate chemistry and carbon sequestration driven by inorganic carbon outwelling from mangroves and saltmarshes. *Nat. Commun.* **14**, 8196 (2023).

8. I. R. Santos, X. Chen, A. L. Lecher, A. H. Sawyer, N. Moosdorf, V. Rodellas, J. Tamborski, H.-M. Cho, N. Dimova, R. Sugimoto, S. Bonaglia, H. Li, M.-C. Hajati, L. Li, Submarine groundwater discharge impacts on coastal nutrient biogeochemistry. *Nat. Rev. Earth Environ.* **2**, 307–323 (2021).

9. A. H. Sawyer, C. H. David, J. S. Famiglietti, Continental patterns of submarine groundwater discharge reveal coastal vulnerabilities. *Science* **353**, 705–707 (2016).

10. E. Luijendijk, T. Gleeson, N. Moosdorf, Fresh groundwater discharge insignificant for the world's oceans but important for coastal ecosystems. *Nat. Commun.* **11**, 1260 (2020).

11. W. S. Moore, The effect of submarine groundwater discharge on the ocean. *Ann. Rev. Mar. Sci.* **2**, 59–88 (2010).

12. J. J. Tamborski, J. K. Cochran, H. J. Bokuniewicz, Submarine groundwater discharge driven nitrogen fluxes to Long Island Sound, NY: Terrestrial vs. marine sources. *Geochim. Cosmochim. Acta* **218**, 40–57 (2017).

13. C. E. Robinson, P. Xin, I. R. Santos, M. A. Charette, L. Li, D. A. Barry, Groundwater dynamics in subterranean estuaries of coastal unconfined aquifers: Controls on submarine groundwater discharge and chemical inputs to the ocean. *Adv. Water Resour.* **115**, 315–331 (2018).

14. A. Cabral, R. Sugimoto, M. Taniguchi, D. Tait, T. Nakajima, H. Honda, I. R. Santos, Fresh and saline submarine groundwater discharge as sources of carbon and nutrients to the Japan Sea. *Mar. Chem.* **249**, 104209 (2023).

15. J. Garcia-Orellana, V. Rodellas, J. Tamborski, M. Diego-Feliu, P. van Beek, Y. Weinstein, M. Charette, A. Alorda-Kleinglass, H. A. Michael, T. Stieglitz, J. Scholten, Radium isotopes as submarine groundwater discharge (SGD) tracers: Review and recommendations. *Earth Sci. Rev.* **220**, 103681 (2021).

16. W. S. Moore, J. L. Sarmiento, R. M. Key, Submarine groundwater discharge revealed by Ra distribution in the upper Atlantic Ocean. *Nat. Geosci.* **1**, 309–311 (2008).

17. W. C. Burnett, H. Bokuniewicz, M. Huettel, W. S. Moore, M. Taniguchi, Groundwater and pore water inputs to the coastal zone. *Biogeochemistry* **66**, 3–33 (2003).

18. S. J. Wilson, A. Moody, T. M. Kenzie, M. B. Cardenas, E. Luijendijk, A. H. Sawyer, A. Wilson, H. A. Michael, B. Xu, K. L. Knee, H.-M. Cho, Y. Weinstein, A. Paytan, N. Moosdorf, C.-T. A. Chen, M. Beck, C. Lopez, D. Murgulet, G. Kim, M. A. Charette, H. Waska, J. S. P. Ibánhez, G. Chaillou, T. Oehler, S.-i. Onodera, M. Saito, V. Rodellas, N. Dimova, D. Montiel, H. Dulai, C. Richardson, J. Du, E. Petermann, X. Chen, K. L. Davis, S. Lamontagne, R. Sugimoto, G. Wang, H. Li, A. I. Torres, C. Demir, E. Bristol, C. T. Connolly, J. W. McClelland, B. J. Silva, D. Tait, B. S. K. Kumar, R. Viswanadham, V. V. S. S. Sarma, E. Silva-Filho, A. Shiller, A. Lecher, J. Tamborski, H. Bokuniewicz, C. Rocha, A. Reckhardt, M. E. Böttcher, S. Jiang, T. Stieglitz, H. G. V. Gbewezoun, C. Charbonnier, P. Anschutz, L. M. Hernández-Terrones, S. Babu, B. Szymczycha, M. Sadat-Noori, F. Niencheski, K. Null, C. Tobias, B. Song, I. C. Anderson, I. R. Santos, Global subterranean estuaries modify groundwater nutrient loading to the ocean. *Limnol. Oceanogr. Lett.* **9**, 411–422 (2024).

19. M. A. Goñi, L. R. Gardner, Seasonal dynamics in dissolved organic carbon concentrations in a coastal water-table aquifer at the forest-marsh interface. *Aquat. Geochem.* **9**, 209–232 (2003).

20. S. Bouillon, J. J. Middelburg, F. Dehairs, A. V. Borges, G. Abril, M. R. Flindt, S. Ulomi, E. Kristensen, Importance of intertidal sediment processes and porewater exchange on the water column biogeochemistry in a pristine mangrove creek (Ras Dege, Tanzania). *Biogeosciences* **4**, 311–322 (2007).

21. M. Call, I. R. Santos, T. Dittmar, C. E. de Rezende, N. E. Asp, D. T. Maher, High pore-water derived CO_2 and CH_4 emissions from a macro-tidal mangrove creek in the Amazon region. *Geochim. Cosmochim. Acta* **247**, 106–120 (2019).

22. W.-J. Cai, M. Dai, Y. Wang, Air-sea exchange of carbon dioxide in ocean margins: A province-based synthesis. *Geophys. Res. Lett.* **33**, L12603 (2006).

23. W. J. Cai, Y. C. Wang, J. Krest, W. S. Moore, The geochemistry of dissolved inorganic carbon in a surficial groundwater aquifer in North Inlet, South Carolina, and the carbon fluxes to the coastal ocean. *Geochim. Cosmochim. Acta* **67**, 631–639 (2003).

24. J. Lee, G. Kim, Dependence of coastal water pH increases on submarine groundwater discharge off a volcanic island. *Estuar. Coast. Shelf Sci.* **163**, 15–21 (2015).

25. E. Y. Kwon, G. Kim, F. Primeau, W. S. Moore, H.-M. Cho, T. DeVries, J. L. Sarmiento, M. A. Charette, Y.-K. Cho, Global estimate of submarine groundwater discharge based on an observationally constrained radium isotope model. *Geophys. Res. Lett.* **41**, 8438–8444 (2014).

26. M. A. Charette, W. S. Moore, W. C. Burnett, Uranium- and thorium-series nuclides as tracers of submarine groundwater discharge. *Radioact. Environ.* **13**, 155–191 (2008).

27. D. Adyasari, N. T. Dimova, H. Dulai, B. S. Gilfedder, I. Cartwright, T. McKenzie, P. Fuleky, Radon-222 as a groundwater discharge tracer to surface waters. *Earth Sci. Rev.* **238**, 104321 (2023).

28. I. R. Santos, D. T. Maher, B. D. Eyre, Coupling automated radon and carbon dioxide measurements in coastal waters. *Environ. Sci. Technol.* **46**, 7685–7691 (2012).

29. L. C. Jeffrey, I. R. Santos, D. R. Tait, U. Makings, D. T. Maher, Seasonal drivers of carbon dioxide dynamics in a hydrologically modified subtropical tidal river and estuary (Caboolture River, Australia). *J. Geophys. Res. Biogeo.* **123**, 1827–1849 (2018).

30. K. Davis, I. R. Santos, A. K. Perkins, J. R. Webb, J. Gleeson, Altered groundwater discharge and associated carbon fluxes in a wetland-drained coastal canal. *Estuar. Coast. Shelf Sci.* **235**, 106567 (2020).

31. L. C. Jeffrey, D. T. Maher, I. R. Santos, A. McMahon, D. R. Tait, Groundwater, acid and carbon dioxide dynamics along a coastal wetland lake and estuary continuum. *Estuaries Coast* **39**, 1325–1344 (2016).

32. X. Chen, I. R. Santos, M. Call, G. M. S. Reithmaier, D. Maher, C. Holloway, P. D. Wadnerkar, P. Gómez-Alvarez, C. J. Sanders, L. Li, The mangrove CO_2 pump: Tidally driven pore-water exchange. *Limnol. Oceanogr.* **66**, 1563–1577 (2021).

33. K.-K. Liu, L. Atkinson, R. Quiñones, L. Talaue-McManus, *Carbon and Nutrient Fluxes in Continental Margins: A Global Synthesis* (Springer Science & Business Media, 2010).

34. D. T. Maher, I. R. Santos, L. Golsby-Smith, J. Gleeson, B. D. Eyre, Groundwater-derived dissolved inorganic and organic carbon exports from a mangrove tidal creek: The missing mangrove carbon sink? *Limnol. Oceanogr.* **58**, 475–488 (2013).

35. K. D. Kroeger, P. W. Swarzenski, W. J. Greenwood, C. Reich, Submarine groundwater discharge to Tampa Bay: Nutrient fluxes and biogeochemistry of the coastal aquifer. *Mar. Chem.* **104**, 85–97 (2007).

36. D. Montiel, A. F. Lamore, J. Stewart, W. J. Lambert, J. Honeck, Y. Lu, O. Warren, D. Adyasari, N. Moosdorf, N. Dimova, Natural groundwater nutrient fluxes exceed anthropogenic inputs in an ecologically impacted estuary: Lessons learned from Mobile Bay, Alabama. *Biogeochemistry* **145**, 1–33 (2019).

37. W. S. Moore, J. O. Blanton, S. B. Joye, Estimates of flushing times, submarine groundwater discharge, and nutrient fluxes to Okatee Estuary, South Carolina. *J. Geophys. Res. Oceans* **111**, C09006 (2006).

38. B. I. McNeil, T. P. Sasse, Future ocean hypercapnia driven by anthropogenic amplification of the natural CO_2 cycle. *Nature* **529**, 383–386 (2016).

39. D. T. Maher, M. Call, P. Macklin, J. R. Webb, I. R. Santos, Hydrological versus biological drivers of nutrient and carbon dioxide dynamics in a coastal lagoon. *Estuaries Coast* **42**, 1015–1031 (2019).

40. W. S. Moore, C. Benitez-Nelson, C. Schutte, A. Moody, A. Shiller, R. J. Sibert, S. Joye, SGD-OD: Investigating the potential oxygen demand of submarine groundwater discharge in coastal systems. *Sci. Rep.* **14**, 9249 (2024).

41. I. R. Santos, R. N. Glud, D. Maher, D. Erler, B. D. Eyre, Diel coral reef acidification driven by porewater advection in permeable carbonate sands, Heron Island Great Barrier Reef. *Geophys. Res. Lett.* **38**, L03604 (2011).

42. L. Keppler, P. Landschützer, S. K. Lauvset, N. Gruber, Recent trends and variability in the oceanic storage of dissolved inorganic carbon. *Global Biogeochem. Cycles* **37**, e2022GB007677 (2023).

43. P. Xin, A. Wilson, C. Shen, Z. Ge, K. B. Moffett, I. R. Santos, X. Chen, X. Xu, Y. Y. Yau, W. Moore, Surface water and groundwater interactions in salt marshes and their impact on plant ecology and coastal biogeochemistry. *Rev. Geophys.* **60**, e2021RG000740 (2022).

44. M. Taniguchi, Tidal effects on submarine groundwater discharge into the ocean. *Geophys. Res. Lett.* **29**, 2-1–2-3 (2002).

45. X. Li, B. X. Hu, W. C. Burnett, I. R. Santos, J. P. Chanton, Submarine ground water discharge driven by tidal pumping in a heterogeneous aquifer. *Groundwater* **47**, 558–568 (2009).

46. G. Kim, D.-W. Hwang, Tidal pumping of groundwater into the coastal ocean revealed from submarine ^{222}Rn and CH_4 monitoring. *Geophys. Res. Lett.* **29**, 23-1–23-4 (2002).

47. I. R. Santos, W. C. Burnett, J. Chanton, N. Dimova, R. N. Peterson, Land or ocean?: Assessing the driving forces of submarine groundwater discharge at a coastal site in the Gulf of Mexico. *J. Geophys. Res. Oceans* **114**, C04012 (2009).

48. A. M. Wilson, T. B. Evans, W. S. Moore, C. A. Schutte, S. B. Joye, What time scales are important for monitoring tidally influenced submarine groundwater discharge? Insights from a salt marsh. *Water Resour. Res.* **51**, 4198–4207 (2015).

49. P. Xin, L. R. Yuan, L. Li, D. A. Barry, Tidally driven multiscale pore water flow in a creek-marsh system. *Water Resour. Res.* **47**, W07534 (2011).

50. I. R. Santos, D. T. Maher, R. Larkin, J. R. Webb, C. J. Sanders, Carbon outwelling and outgassing vs. burial in an estuarine tidal creek surrounded by mangrove and saltmarsh wetlands. *Limnol. Oceanogr.* **64**, 996–1013 (2019).

51. A. Cabral, Y. Y. Yau, G. M. Reithmaier, L. C. Cotovicz Jr., J. Barreira, G. Broström, B. Viana, A. L. Fonseca, I. R. Santos, Tidally driven porewater exchange and diel cycles control CO_2

fluxes in mangroves on local and global scales. *Geochim. Cosmochim. Acta* **374**, 121–135 (2024).

52. L. C. Cotovicz Jr., G. Abril, C. J. Sanders, D. R. Tait, D. T. Maher, J. Z. Sippo, C. Holloway, Y. Y. Y. Yau, I. R. Santos, Methane oxidation minimizes emissions and offsets to carbon burial in mangroves. *Nat. Clim. Chang.* **14**, 275–281 (2024).

53. R. M. Cory, C. P. Ward, B. C. Crump, G. W. Kling, Sunlight controls water column processing of carbon in arctic fresh waters. *Science* **345**, 925–928 (2014).

54. L. J. Tranvik, J. A. Downing, J. B. Cotner, S. A. Loiselle, R. G. Striegl, T. J. Ballatore, P. Dillon, K. Finlay, K. Fortino, L. B. Knoll, P. L. Kortelainen, T. Kutser, S. Larsen, I. Laurion, D. M. Leech, S. L. McCallister, D. M. Mc Knight, J. M. Melack, E. Overholt, J. A. Porter, Y. Prairie, W. H. Renwick, F. Roland, B. S. Sherman, D. W. Schindler, S. Sobek, A. Tremblay, M. J. Vanni, A. M. Verschoor, E. von Wachenfeldt, G. A. Weyhenmeyer, Lakes and reservoirs as regulators of carbon cycling and climate. *Limnol. Oceanogr.* **54**, 2298–2314 (2009).

55. K. K. Yates, C. Dufore, N. Smiley, C. Jackson, R. B. Halley, Diurnal variation of oxygen and carbonate system parameters in Tampa Bay and Florida Bay. *Mar. Chem.* **104**, 110–124 (2007).

56. V. Rodellas, T. C. Stieglitz, J. J. Tamborski, P. van Beek, A. Andrisoa, P. G. Cook, Conceptual uncertainties in groundwater and porewater fluxes estimated by radon and radium mass balances. *Limnol. Oceanogr.* **66**, 1237–1255 (2021).

57. K. M. Coluccio, I. R. Santos, L. C. Jeffrey, L. K. Morgan, Groundwater discharge rates and uncertainties in a coastal lagoon using a radon mass balance. *J. Hydrol.* **598**, 126436 (2021).

58. S. B. Joye, D. A. Bronk, D. J. Koopmans, W. S. Moore, "Evaluating the potential importance of groundwater-derived carbon, nitrogen, and phosphorus inputs to South Carolina and Georgia coastal ecosystems" in *Changing Land Use Patterns in the Coastal Zone: Managing Environmental Quality in Rapidly Developing Regions* (Springer, 2006), pp. 139–178.

59. C. M. Duarte, N. Marbà, E. Gacia, J. W. Fourqurean, J. Beggins, C. Barrón, E. T. Apostolaki, Seagrass community metabolism: Assessing the carbon sink capacity of seagrass meadows. *Global Biogeochem. Cycles* **24**, GB4032 (2010).

60. R. E. Correa, K. Xiao, S. R. Conrad, P. D. Wadnerkar, A. M. Wilson, C. J. Sanders, I. R. Santos, Groundwater carbon exports exceed sediment carbon burial in a salt marsh. *Estuaries Coast* **45**, 1545–1561 (2022).

61. H. A. McGowan, M. C. MacKellar, M. A. Gray, Direct measurements of air-sea CO_2 exchange over a coral reef. *Geophys. Res. Lett.* **43**, 4602–4608 (2016).

62. M. Sadat-Noori, D. R. Tait, D. T. Maher, C. Holloway, I. R. Santos, Greenhouse gases and submarine groundwater discharge in a Sydney Harbour embayment (Australia). *Estuar. Coast. Shelf Sci.* **207**, 499–509 (2018).

63. I. R. Santos, M. Beck, H.-J. Brumsack, D. T. Maher, T. Dittmar, H. Waska, B. Schnetger, Porewater exchange as a driver of carbon dynamics across a terrestrial-marine transect: Insights from coupled ^{222}Rn and pCO_2 observations in the German Wadden Sea. *Mar. Chem.* **171**, 10–20 (2015).

64. J. A. Rosentreter, D. Maher, D. Erler, R. Murray, B. Eyre, Seasonal and temporal CO_2 dynamics in three tropical mangrove creeks—A revision of global mangrove CO_2 emissions. *Geochim. Cosmochim. Acta* **222**, 729–745 (2018).

65. D. M. Alongi, Carbon cycling and storage in mangrove forests. *Ann. Rev. Mar. Sci.* **6**, 195–219 (2014).

66. S. Bouillon, A. V. Borges, E. Castañeda-Moya, K. Diele, T. Dittmar, N. C. Duke, E. Kristensen, S. Y. Lee, C. Marchand, J. J. Middelburg, V. H. Rivera-Monroy, T. J. Smith III, R. R. Twilley, Mangrove production and carbon sinks: A revision of global budget. *Global Biogeochem. Cycles* **22**, GB2013 (2008).

67. A. V. Borges, S. Djenidi, G. Lacroix, J. Théate, B. Delille, M. Frankignoulle, Atmospheric CO_2 flux from mangrove surrounding waters. *Geophys. Res. Lett.* **30**, 1558 (2003).

68. J. J. Cole, Y. T. Prairie, N. F. Caraco, W. H. M. Dowell, L. J. Tranvik, R. G. Striegl, C. M. Duarte, P. Kortelainen, J. A. Downing, J. J. Middelburg, J. Melack, Plumbing the global carbon cycle: Integrating inland waters into the terrestrial carbon budget. *Ecosystems* **10**, 172–185 (2007).

69. J. J. Middelburg, K. Soetaert, M. Hagens, Ocean alkalinity, buffering and biogeochemical processes. *Rev. Geophys.* **58**, e2019RG000681 (2020).

70. P. Kortelainen, M. Rantakari, J. T. Huttunen, T. Mattsson, J. Alm, S. Juutinen, T. Larmola, J. Silvola, P. J. Martikainen, Sediment respiration and lake trophic state are important predictors of large CO_2 evasion from small boreal lakes. *Glob. Chang. Biol.* **12**, 1554–1567 (2006).

71. G. Wang, W. Jing, S. Wang, Y. Xu Yi, Z. Wang, Z. Zhang, Q. Li, M. Dai, Coastal acidification induced by tidal-driven submarine groundwater discharge in a coastal coral reef system. *Environ. Sci. Technol.* **48**, 13069–13075 (2014).

72. J. E. Bauer, W.-J. Cai, P. A. Raymond, T. S. Bianchi, C. S. Hopkinson, P. A. Regnier, The changing carbon cycle of the coastal ocean. *Nature* **504**, 61–70 (2013).

73. G. M. S. Reithmaier, D. T. Maher, C. Holloway, R. E. Correa, I. R. Santos, Small wetland-fringed estuaries deliver disproportionately large carbon loads to the ocean. *Limnol. Oceanogr.* **69**, 2229–2242 (2024).

74. C. McCoy, D. Corbett, Review of submarine groundwater discharge (SGD) in coastal zones of the Southeast and Gulf Coast regions of the United States with management implications. *J. Environ. Manage.* **90**, 644–651 (2009).

75. D. Pierrot, C. Neill, K. Sullivan, R. Castle, R. Wanninkhof, H. Lüger, T. Johannessen, A. Olsen, R. A. Feely, C. E. Cosca, Recommendations for autonomous underway pCO_2 measuring systems and data-reduction routines. *Deep Sea Res. 2 Top. Stud. Oceanogr.* **56**, 512–522 (2009).

76. W. Burnett, G. Kim, D. Lane-Smith, A continuous monitor for assessment of ^{222}Rn in the coastal ocean. *J. Radioanal. Nucl. Chem.* **249**, 167–172 (2001).

77. H. Hersbach, B. Bell, P. Berrisford, G. Biavati, A. Horányi, J. Muñoz Sabater, J. Nicolas, C. Peubey, R. Radu, I. Rozum, D. Schepers, A. Simmons, C. Soci, D. Dee, J.-N. Thépaut, ERA5 hourly data on single levels from 1940 to present [Copernicus Climate Change Service (C3S), Climate Data Store (CDS), 2023] (accessed 11 November 2022); https://doi.org/10.24381/cds.adbb2d47.

78. M. A. Charette, M. C. Allen, Precision ground water sampling in coastal aquifers using a direct-push, shielded-screen well-point system. *Groundwater Monit. Remediat.* **26**, 87–93 (2006).

79. J.-M. Lee, G. Kim, A simple and rapid method for analyzing radon in coastal and ground waters using a radon-in-air monitor. *J. Environ Radioact.* **89**, 219–228 (2006).

80. J. R. Gatland, I. R. Santos, D. T. Maher, T. Duncan, D. V. Erler, Carbon dioxide and methane emissions from an artificially drained coastal wetland during a flood: Implications for wetland global warming potential. *J. Geophys. Res. Biogeosci.* **119**, 1698–1716 (2014).

81. D. Pierrot, D. Lewis, D. Wallace, CO2Sys DOS program developed for CO_2 system calculations (Carbon Dioxide Information Analaysis Center, Oak Ridge National Laboratory, 2006).

82. C. Mehrbach, C. H. Culberson, J. E. Hawley, R. M. Pytkowicx, Measurement of the apparent dissociation constants of carbonic acid in seawater at atmospheric pressure 1. *Limnol. Oceanogr.* **18**, 897–907 (1973).

83. A. G. Dickson, F. J. Millero, A comparison of the equilibrium constants for the dissociation of carbonic acid in seawater media. *Deep Sea Res. A Oceanogr. Res. Pap.* **34**, 1733–1743 (1987).

84. R. Wanninkhof, Relationship between wind speed and gas exchange over the ocean. *J. Geophys. Res. Oceans* **97**, 7373–7382 (1992).

85. R. F. Weiss, Carbon dioxide in water and seawater: The solubility of a non-ideal gas. *Mar. Chem.* **2**, 203–215 (1974).

86. A. V. Borges, B. Delille, L. S. Schiettecatte, F. Gazeau, G. Abril, M. Frankignoulle, Gas transfer velocities of CO_2 in three European estuaries (Randers Fjord, Scheldt, and Thames). *Limnol. Oceanogr.* **49**, 1630–1641 (2004).

87. W. C. Burnett, H. Dulaiova, Estimating the dynamics of groundwater input into the coastal zone via continuous radon-222 measurements. *J. Environ. Radioact.* **69**, 21–35 (2003).

88. S. MacIntyre, Trace gas exchange across the air-sea interface in fresh water and coastal marine environments. *Biog. Trace Gases: Meas. Emiss. Soil Water* , 52–97 (1995).

89. P. Regier, H. Briceño, J. N. Boyer, Analyzing and comparing complex environmental time series using a cumulative sums approach. *MethodsX* **6**, 779–787 (2019).

90. M. Call, D. T. Maher, I. R. Santos, S. Ruiz-Halpern, P. Mangion, C. J. Sanders, D. V. Erler, J. M. Oakes, J. Rosentreter, R. Murray, B. D. Eyre, Spatial and temporal variability of carbon dioxide and methane fluxes over semi-diurnal and spring–neap–spring timescales in a mangrove creek. *Geochim. Cosmochim. Acta* **150**, 211–225 (2015).

91. T. Cyronak, I. R. Santos, D. V. Erler, D. T. Maher, B. D. Eyre, Drivers of pCO_2 variability in two contrasting coral reef lagoons: The influence of submarine groundwater discharge. *Global Biogeochem. Cycles* **28**, 398–414 (2014).

92. C. O'Reilly, I. R. Santos, T. Cyronak, A. McMahon, D. T. Maher, Nitrous oxide and methane dynamics in a coral reef lagoon driven by pore water exchange: Insights from automated high-frequency observations. *Geophys. Res. Lett.* **42**, 2885–2892 (2015).

93. A. S. Tomer, T. McKenzie, C. Majtenyi-Hill, A. Cabral, Y. Y. Y. Yau, M. Call, X. Chen, R. E. Correa, K. Davis, L. Jeffrey, M. Sadar-Noori, D. Tait, J. Webb, D. T. Maher, L. Henriksson, S. Bonaglia, S. Zhao, M. B. Cardenas, I. R. Santos, Global Data for SGD and CO2, version 1, Zenodo (2024); https://doi.org/10.5281/zenodo.10491455.

94. J. P. Archibald, I. R. Santos, K. L. Davis, Diel versus tidal cycles of chromophoric dissolved organic matter (CDOM) and radon in a coral reef in the Great Barrier Reef. *Reg. Stud. Mar. Sci.* **29**, 100659 (2019).

95. R. E. Correa, M. B. Cardenas, R. S. Rodolfo, M. R. Lapus, K. L. Davis, A. B. Giles, J. C. Fullon, M.-C. Hajati, N. Moosdorf, C. J. Sanders, I. R. Santos, Submarine groundwater discharge releases CO_2 to a coral reef. *ACS ES T Water* **1**, 1756–1764 (2021).

96. M. Call, C. J. Sanders, P. A. Macklin, I. R. Santos, D. T. Maher, Carbon outwelling and emissions from two contrasting mangrove creeks during the monsoon storm season in Palau, Micronesia. *Estuar. Coast. Shelf Sci.* **218**, 340–348 (2019).

97. P. D. Wadnerkar, B. Batsaikhan, S. R. Conrad, K. Davis, R. E. Correa, C. Holloway, S. A. White, C. J. Sanders, I. R. Santos, Contrasting radium-derived groundwater exchange and nutrient lateral fluxes in a natural mangrove versus an artificial canal. *Estuaries Coast.* **44**, 123–136 (2021).

98. A. K. Perkins, I. R. Santos, M. Sadat-Noori, J. R. Gatland, D. T. Maher, Groundwater seepage as a driver of CO_2 evasion in a coastal lake (Lake Ainsworth, NSW, Australia). *Environ. Earth Sci.* **74**, 779–792 (2015).

99. Y. Y. Y. Yau, G. Reithmaier, C. Majtényi-Hill, O. Serrano, N. Piñeiro-Juncal, M. Dahl, M. A. Mateo, S. Bonaglia, I. R. Santos, Methane emissions in seagrass meadows as a small offset to carbon sequestration. *J. Geophys. Res. Biogeosci.* **128**, e2022JG007295 (2023).

100. L. Henriksson, Y. Y. Y. Yau, C. Majtényi-Hill, W. Ljungberg, A. S. Tomer, S. Zhao, F. Wang, A. Cabral, M. Asplund, I. R. Santos, Kristineberg Seagrass CH4 & CO_2 Timeseries Data, version 2, Zenodo (2024); https://doi.org/10.5281/zenodo.11493204.

101. M. Sadat-Noori, I. R. Santos, D. R. Tait, D. T. Maher, Fresh meteoric versus recirculated saline groundwater nutrient inputs into a subtropical estuary. *Sci. Total Environ.* **566-567**, 1440–1453 (2016).

102. X. Chen, I. R. Santos, D. Hu, L. Zhan, Y. Zhang, Z. Zhao, S. Hu, L. Li, Pore-water exchange flushes blue carbon from intertidal saltmarsh sediments into the sea. *Limnol. Oceanogr. Lett.* **7**, 312–320 (2022).

103. R. M. Diggle, D. R. Tait, D. T. Maher, X. Huggins, I. R. Santos, The role of porewater exchange as a driver of CO_2 flux to the atmosphere in a temperate estuary (Squamish, Canada). *Environ. Earth Sci.* **78**, 336 (2019).

104. M. L. Atkins, I. R. Santos, S. Ruiz-Halpern, D. T. Maher, Carbon dioxide dynamics driven by groundwater discharge in a coastal floodplain creek. *J. Hydrol.* **493**, 30–42 (2013).
